# Essential Components from Plant Source Oils: A Review on Extraction, Detection, Identification, and Quantification

**DOI:** 10.3390/molecules28196881

**Published:** 2023-09-29

**Authors:** Muhammad Abdul Rahim, Hudda Ayub, Aqeela Sehrish, Saadia Ambreen, Faima Atta Khan, Nizwa Itrat, Anum Nazir, Aurbab Shoukat, Amna Shoukat, Afaf Ejaz, Fatih Özogul, Elena Bartkiene, João Miguel Rocha

**Affiliations:** 1 Department of Food Science, Faculty of Life Sciences, Government College University, Faisalabad 38000, Pakistan; faimaft01@gmail.com (F.A.K.); afafejaz786@gmail.com (A.E.); 2 National Institute of Food Science & Technology, University of Agriculture, Faisalabad 38000, Pakistan; huddaayub141@gmail.com (H.A.); aaniq.ks786@gmail.com (A.S.); amnashaukat550@gmail.com (A.S.); 3 Department of Plant and Soil Science, Texas Tech University, Lubbock, TX 79409, USA; aqeela.sehrish@ttu.edu; 4 University Institute of Food Science and Technology, The University of Lahore, Lahore 54590, Pakistan; saadia.ambreen@uifst.uol.edu.pk; 5 Department of Nutrition and Dietetics, The University of Faisalabad, Faisalabad 38000, Pakistan; nizwaqamar@gmail.com (N.I.); anum.nazir@tuf.edu.pk (A.N.); 6 Department of Seafood Processing Technology, Faculty of Fisheries, Cukurova University, Balcali, Adana 01330, Türkiye; fozogul@cu.edu.tr; 7 Biotechnology Research and Application Center, Cukurova University, Balcali, Adana 01330, Türkiye; 8 Department of Food Safety and Quality, Faculty of Veterinary, Lithuanian University of Health Sciences, Tilzes Str. 18, LT-47181 Kaunas, Lithuania; elena.bartkiene@lsmu.lt; 9 Faculty of Animal Sciences, Institute of Animal Rearing Technologies, Lithuanian University of Health Sciences, Tilzes Str. 18, LT-47181 Kaunas, Lithuania; 10 Universidade Católica Portuguesa, CBQF—Centro de Biotecnologia e Química Fina—Laboratório Associado, Escola Superior de Biotecnologia, Rua Diogo Botelho 1327, 4169-005 Porto, Portugal; 11 LEPABE—Laboratory for Process Engineering, Environment, Biotechnology and Energy, Faculty of Engineering, University of Porto, Rua Dr. Roberto Frias, s/n, 4200-465 Porto, Portugal; 12 ALiCE—Associate Laboratory in Chemical Engineering, Faculty of Engineering, University of Porto, Rua Dr. Roberto Frias, s/n, 4200-465 Porto, Portugal

**Keywords:** seed oils, extraction, identification and quantification techniques, applications

## Abstract

Oils derived from plant sources, mainly fixed oils from seeds and essential oil from other parts of the plant, are gaining interest as they are the rich source of beneficial compounds that possess potential applications in different industries due to their preventive and therapeutic actions. The essential oils are used in food, medicine, cosmetics, and agriculture industries as they possess antimicrobial, anticarcinogenic, anti-inflammatory and immunomodulatory properties. Plant based oils contain polyphenols, phytochemicals, and bioactive compounds which show high antioxidant activity. The extractions of these oils are a crucial step in terms of the yield and quality attributes of plant oils. This review paper outlines the different modern extraction techniques used for the extraction of different seed oils, including microwave-assisted extraction (MAE), pressurized liquid extraction (PLE), cold-pressed extraction (CPE), ultrasound-assisted extraction (UAE), supercritical-fluid extraction (SFE), enzyme-assisted extraction (EAE), and pulsed electric field-assisted extraction (PEF). For the identification and quantification of essential and bioactive compounds present in seed oils, different modern techniques—such as high-performance liquid chromatography (HPLC), gas chromatography–mass spectrometry (GC-MS), Fourier transform infrared spectroscopy (FTIR), gas chromatography–infrared spectroscopy (GC-IR), atomic fluorescence spectroscopy (AFS), and electron microscopy (EM)—are highlighted in this review along with the beneficial effects of these essential components in different in vivo and in vitro studies and in different applications. The primary goal of this research article is to pique the attention of researchers towards the different sources, potential uses and applications of oils in different industries.

## 1. Overview

Various medicinal and dietary plants have been used since ancient times for several purposes. In recent times, there was an increasing attention and diffusion of plant seed oils for their health benefits and uses in cooking. Such plant seed oil is important because of its beneficial nutrients and essential fatty acids. Plant seeds oils are a healthy addition to the diet as they are rich sources of omega 3, omega 6, and omega 9 fatty acids, they are heart healthy and they improve brain functioning thus promoting human health. Below are some examples of different seed oils which are most commonly used and have high availability. Seed oils have high lipid content which contain a high fatty acid profile and other phytochemicals. Chia (*Salvia hispanica* L.) seeds contains dietary fiber, protein, and fat and are a rich source of the B vitamins, minerals, and phytochemicals but mainly provide unsaturated fatty acids (linoleic acid and α-linolenic acid) [1,2]. Flaxseeds (*Linum usitatissimmum*) are the richest source of α-linolenic acid, lignans, soluble fiber, antioxidants, and high-quality protein. By consuming this seed one may lower both total and low-density lipoprotein (LDL) cholesterol [3]. Pumpkin seeds belong to the Cucurbitaceae family and are tiny and densely packed with beneficial nutrients and nutraceuticals, such as amino acids, phytosterols, unsaturated fatty acids (UFA), phenolic compounds, tocopherols, cucurbitacins, and valuable minerals [4]. Cottonseed quality is determined by its content of mineral and non-mineral nutrients, because of their direct or indirect contribution to protein synthesis, oil, carbohydrates, metabolite synthesis, integrity of cell membranes, and cell wall structure [5]. Coriander (*Coriandrum sativum* L.) seeds contain fixed oils, triglycerides, sugars, proteins, and vitamin C, and are utilized as a seasoning agent [6]. Sunflower (*Helianthus annuus* L.) seeds are a good source of dietary fiber, proteins, and vitamins (E, B, folic acid) [7]. Safflower (*Carthamus tinctorius* L.) seed is primarily grown for its oil. The proteins from the safflower seeds are also of good nutritional quality [8]. Clove (*Syzygium aromaticum* L.) is one of the major vegetal sources of phenolic compounds, containing eugenol [9]. Basil (*Ocimum* L.) seed is used for propagation. Basil seeds contain planteose, mucilage, polysaccharides, and fixed oil [10]. The oil from flaxseed (linseed) (*Linum usitatissimum* L.) is predominantly a source of omega-3 fatty acids (ω-3 fatty acids) [11]. Coconuts, when mature, contain some water and can be used as seed nuts or processed to extract oil from the kernel, charcoal from the hard shell, and the fiber from the husk [12]. Canola seed is rich in omega-3 fatty acids and its oil is an excellent source of α-linolenic acid [13]. Rice (*Oryzae sativa* L.) bran is the outer layer of the rice kernel. Appreciable quantities of antioxidants are present in rice bran and it has been known as a potential source of edible oil [14]. Peanuts (*Arachis hypogaea* L.) are an inexpensive and nutritionally powerful food source for people worldwide [15]. Soybean (*Glycine max* L.) seed comprises high protein content that makes it a valuable source for soy food and feed production [16]. Sesame (*Sesamum indicum* L.) seeds are an important source of oil, protein, carbohydrates, and minerals for human nutrition [17]. Corn (*Zea mays* L.) seeds are made up of an episperm, endosperm, and embryo. Nutritional ingredients in embryos and endosperm—such as protein, fat, and starch—provide the energy for metabolism and respiration [18]. Palms contribute significantly to the global production of vegetable oil [19]. Berry seeds are also rich in oil, with a high content of polyunsaturated fatty acids (PUFA), and are rich in vitamin E, carotenoids, and bioactive compounds with antioxidant activities [20]. Olive oil mostly contains monounsaturated fatty acids (MUFA), with oleic acid being the main fatty acid [21]. Eucalyptus (*Eucapyptus* spp.) contains a high abundance of bioactive secondary metabolites with antiviral and antibacterial effects [22]. Tomato seeds mainly contain protein, phytochemicals, phenolic compounds, flavonoids, unsaponifiable compounds, and antioxidants [23]. Tea seed (*Camellia sinensis kuntze*) contains a large number of saponins and is rich in antioxidants (vitamin E and polyphenols) [24]. Watermelon seeds (*Citrullus lanatus*) have high mineral contents and biologically active compounds like tocopherols, phospholipids, and sterols [25]. The pomegranate (*Punica granatum* L.) has seeds that contain high amounts of lipids, protein, sugars, essential minerals, and some phytoestrogen compounds [26]. Due to the presence of glucosinolates and antioxidants in mustard seeds, they can be used as a source of bioactive compounds [27]. Poppy (*Papaver somniferum*) seeds’ properties and oil yield can vary due to different seeds’ colors and edaphoclimatic conditions [28]. Wheat (*Triticum aestivum*) germ (the embryo) is a concentrated source of antioxidants and a natural source of vitamin E [29]. Okra (*Abelmoschus esculentus*) is a rich source of oil and protein [30]. *Moringa oleifera* (Moringaceae) seeds, or ben seeds, contain a significant amount of oil with a high-quality fatty acid composition [31]. Borneo tallow nut (*Shorea stenoptera*) is mainly used for the oil extracted from its seeds, which is rich in stearic acid [32]. Carob or locust bean gum is extracted from carob (*Ceratonia siliqua* L.) seeds [33]. Perilla seeds (*Fructus perillae*) are a good source of polyunsaturated fatty acids, especially α -linolenic acid [34]. Sylimarin is found in the seeds of *Silybum marianum* L. which are considered a standardized dry extract containing mainly flavonolignans [35]. Black seeds from the Ranunculaceae family are black, bitter and have many different chemical ingredients, including thymoquinones, flavonoids, anthocyanins, alkaloids, and essential fatty acids—particularly linoleic and oleic acid [36]. Avocado (*Persea americana* L.) seed contains starch, high levels of potassium and antioxidants, and is an excellent dietary fiber source [37].

Different oil extraction techniques from plant sources are being practiced and work differently: cold pressing extraction (CPE) requires mechanical power and does not need organic solvents [38]; supercritical-fluid extraction (SFE) uses a supercritical fluid as a solvent; ultrasound-assisted extraction (UAE) involves ultrasonic waves [39], while microwave-assisted extraction (MAE) uses microwave energy [40], and pulsed electric field (PEF) technology uses short electricity pulses [41]; pressurized liquid extraction (PLE) uses conventional solvents under high temperatures and pressure for extraction in a short time [42]; and enzyme-assisted extraction (EAE) involves enzyme hydrolysis that allows the effective extraction and delivery of bioactive compounds [43].

The fatty acid profile, chemical composition, and harmful impurities and adulteration in oils can be detected through various identification and quantification techniques which includes high-performance liquid chromatography (HPLC) [44], gas-chromatography (GC) with mass spectrometry (MS) (GC-MS) [45], gas chromatography–infrared (GC-IR) spectroscopy [46], liquid chromatography–mass spectrometry (LC-MS) [47], Fourier transform infrared (FTIR) spectroscopy [48], gel-permeation chromatography (GPC) [49] and IR spectroscopy (GPC-IR) [50], electron microscopes [51], laser diffraction (LD) [52], and high-resolution mass spectrometry (HRMS) [53,54]. This review compromises different seed oil compositions, characterization of their nutritional profile, identification and quantification techniques and their therapeutic use in food and other industries.

## 2. Different Plant Seeds

### 2.1. Chia Seeds

*Salvia hispanica* commonly known as chia is an annual herbaceous plant, which belongs to the family Lamiaceae, the subfamily Nepetoideae and genus *Salvia* [55]. Chia seed has many nutraceutical advantages and is among the functional foods. According to different studies, chia seeds are found to be very effective in the fight against many diseases [56]. The word chia is derived from the Spanish word “Chien” or “Chian” meaning “oily” [57]. While it ranges from a gorgeously marbled grey color to a dark brown color, seeds have been reported for their beneficial health protection against a variety of metabolic illnesses, and all of its leaves, blossoms, and seeds can be utilized [58]. Because of its highly balanced nutritional composition of omega-3 polyunsaturated fatty acids, fiber, proteins, vitamins, minerals, and antioxidants, chia seeds are considered as plant-based nutraceuticals and have attracted the interest of nutritionists. The greater concentrations of alpha-linolenic (α-linolenic) acid (ALA) found in chia seeds have been linked to hepato- and cardioprotective effects [59] Its dietary fiber helps the digestive system, and research has shown that it may have antidiabetic and anticancer properties. Given all these nutritional advantages, it may be regarded as a new “superfood” [60].

### 2.2. Flaxseed

Flaxseed, which is often known as Linseed, is a crop that is widely grown for both fiber and food. The four most popular preparations of flaxseed include flaxseed oil, ground flaxseed, partly defatted flaxseed meal, and whole flaxseed, all of which are suitable for human consumption [3]. Alpha-linolenic acid, fiber, and lignans are key bioactive components in flaxseeds. With a protein level of up to 40%, flaxseed meal, a principal by-product of the flaxseed oil extraction process, has been well-researched as a rich protein source. Physiological features of flaxseed protein (FP) and FP-derived peptides from flaxseed meals include antimicrobial activity, angiotensin-converting enzyme (ACE) inhibition, antidiabetic impact, and antioxidant capacity [61].

### 2.3. Pumpkin Seed

The Cucurbitaceae family includes pumpkins with oily seeds. Even though several kinds are farmed all over the world, *Cucurbita maxima*, *Cucurbita pepo*, *Cucurbita stilbo*, *Cucurbita moschata*, and *Cucurbita mixta* are the most economically significant species [62]. Pumpkins are grown throughout the world for a variety of functions, including commercial, ornamental, and agricultural usage [63]. Like other seeds, pumpkin seeds are abundant in useful elements. Triterpenoids, phytosterols, phenolic compounds, and their derivatives, unsaturated fatty acids, coumarins, vitamin E (tocopherols), flavonoids, proteins, provitamins, carotenoids, pigments, squalene, saponins, and pyrazine are all abundant in the seeds [4]. Additionally, pumpkin seeds are an excellent source of minor minerals including zinc, manganese, iron, calcium, sodium, and copper, as well as magnesium, potassium, and phosphorus. Some of these bioactive compounds can confer physiological advantages, promote wellbeing, and lower the risk of non-communicable diseases like tumors and microbial infections, hyperglycemia and diabetes oxidative stress-related complications, and prostate disorders [64,65].

### 2.4. Coriander Seed

A tiny, annual plant related to parsley in the Apiaceae family, coriander is typically referred to as a spice. The flavor of coriander seeds is moderate, sweet, somewhat spicy, and has citrus and sage undertones. Crude protein, fat, crude fiber, and ash levels range from 10.9 to 20.8%, 18.1 to 19.5%, 27.3 to 28.8%, and 4.8 to 5.9%, respectively, among other elements. Linalool, which ranges from 50 to 70%, is the primary component of the oil found in coriander seeds [66].

The plant is frequently planted for its seed. The monoterpenoid linalool [67] is the main component of the oil and the seeds contain up to 1% (weight percentage, wt./wt.) of it. The seed is mostly known for its usage in medicine as a remedy for dyspepsia, worms, rheumatism, and joint discomfort. Due to its usage as a flavoring component in cosmetics, fragrances, and culinary goods, the seed also has significant commercial value [68].

### 2.5. Sunflower Seed

Common sunflower, a member of the family Asteraceae, is commercially cultivated all over the world and confers a lot of advantages to human health. Sunflower seeds are collected mostly for oil purposes, coming in fourth place globally [7.7% of 185.9 million tons (Mt) of oil in the year 2012] after oilseed rape (12.9%) and oilseed palm (28.8%), despite being used as a snack, salad garnish, and in certain baked items [69]. The antioxidant, antibacterial, anti-hypertensive, anti-inflammatory, wound-healing, and cardiovascular advantages of the sunflower seed and sprout may be found in flavonoids, vitamins, polyunsaturated fatty acids, and phenolic compounds [69]. Sunflower seed has an oil content of 35–42%, is naturally rich in linoleic acid (57–70%), and has modest levels of oleic acid (20–25%) [70]. According to the undertaken research, sunflower oil has antioxidant qualities and may lower LDL and total cholesterol [71].

### 2.6. Safflower Seed

The family Asteraceae, which has its roots extended to the Middle East, includes the oldest crop in the world, *Carthamus tinctorius* L., which is often known as safflower. Safflowers have an extensive history of usage as a plant for medicine and as a natural source of dye for clothing and food coloring. The whole seeds of safflower with typical hull types contain close to 28–31% oil of very high quality, 13.0–14.9% proteins, 5.1–8.1% moisture, 31.9–40.3% crude fiber, and 2.3–6.8% ash. It has also been demonstrated that the seed oil is particularly rich in linoleic acid (70–87%), a type of polyunsaturated essential fatty acid [72]. According to Cosge et al. (2007), the major constituents of safflower oil are palmitic, stearic, oleic, and linoleic acids, with oleic and linoleic acids making up the great majority. Safflower seed oil also has high vitamin E activity and is thought to be a significant source of α-tocopherols [73]. Safflower oil, which has a mild flavor and a light hue, is frequently used in cooking, margarine production, and salad dressing. Safflower seed oil is a top-notch health product that has been shown to prevent and cure coronary heart disease (CHD), arteriosclerosis, and hyperlipemia [74].

### 2.7. Clove

The fragrant dried flower buds known as cloves (*Syzygium aromaticum*) are from a tree in the Myrtaceae family. Cloves are a common spice used around the world. An evergreen tree’s bloom buds are used to create the flavor known as cloves. The use of clove in medicine and as a food preservative is widespread [75]. Eugenol is a substance found in cloves. There is evidence that it functions as a natural antioxidant. Cloves composition is as follows: moisture (29.47%), fiber (14.37%), ash (5.29%), protein (6.91%), fat (5.86%), and carbs (32.1%) [9]. Eugenol is found in concentrations between 72 and 91% of the oil extracted from the clove. Clove oil contains Beta-caryophyllene, acetyl eugenol, methyl salicylate, vanillin, gallotannic acid, tannins, triterpenoid, kaempferol, eugenin, and oleanolic acid. The numerous medical applications for cloves include pain relief, nausea and vomiting management, and digestive enhancement. It works as an antibacterial agent against bacteria and fungi, inhibits the growth of internal parasites, and induces uterine contractions [76]. Clove is a powerful remedy for gastrointestinal spasms, bloating, and flatulence in conventional medicine [77].

### 2.8. Basil Seed

*Ocimum basilicum* L., commonly referred to as basil or sweet basil, belongs to the Labiatae family and is an annual herb known for its pleasant spiciness. The name “basil” originates from the Greek word “Basileus,” which means “Royal” or “King.” [78] The emulsifying, thickening, foaming, stabilizing, viscosity, and gelling properties of basil seed mucilage have been thoroughly investigated [79]. In terms of phenolic acid content, flavonoids and the amounts of Vicentine, orientine, and rosmarinic acids are remarkable in basil seeds [80]. Due to its versatile applications in culinary, medical, cosmetic, and pharmaceutical fields, it is termed as the King of herbs.” Basil seeds, rich in dietary fiber, are used to enhance the properties of fruit-based beverages. These seeds possess potential as functional ingredients due to their mucilage, which exhibits qualities like emulsification, foaming, thickening, and gelling [81]. Although basil seeds have shown promising health benefits such as antidiabetic, antibacterial, antioxidant, and anticancer properties, they are not commonly consumed as a food source. The primary phenolic components of basil are phenolic acids and flavonol glycosides. Additionally, basil contains various fatty acids, including stearic acid, oleic acid, palmitic acid, linoleic acid, myristic acid, α-linolenic acid, capric acid, lauric acid, and arachidonic acid [82]. Notably, basil’s antioxidant content is represented by compounds like caffeic, vanillic, and rosmarinic acids, along with quercetin, rutin, apigenin, chlorogenic acid, and p-hydroxybenzoic acid. Basil oils consist of components like α-pinene, β-pinene, methyl chavicol, 1,8 cineole, linalool, ocimene, borneol, geraniol, β-caryophyllene, n-cinnamate, and eugenol. Among these, eugenol, chavicol, and various terpenoids are the most notable [83,84].

### 2.9. Coconut

The coconut tree (*Cocos nucifera* L.) is one of the members of the palm tree family. An important perennial crop of the humid tropics, the coconut is a multifunctional crop that provides food, edible oil, a cool beverage, fiber (coir), fodder, and fuel [85]. Coconut oil is widely utilized in both food and industry. The oil has a high concentration of medium-chain fatty acids (MCFA) and is easily digestible [86]. Lauric acid makes up the majority of the fatty acids, with a proportion ranging from 46 to 48% [87]. Research has revealed that between 60 and 63% of the medium-chain fatty acids are present. The predominant triacylglycerols (TAG) in the coconut oil samples are 22–25% Lauric acid, 14–16% capric acid, and 19–21% myristic acids [88]. Protocatechuic, vanillic, caffeic, syringic, ferulic, and p-coumaric acids were among the phenolic acids found in coconut oil. Phenolic compounds are responsible for some of the antioxidant action of coconut oil. Additionally, caffeic, p-coumaric, and ferulic acids as well as catechin are said to be present in both conventional and commercial coconut oil [89].

### 2.10. Canola Seed

The canola oilseed is a member of the Brassica family of rapeseeds. Its name, which is a trademark, is a contraction of “Canada” and “ola,” which stands for low-acid oil [90]. One of the most significant sources of edible vegetable oils worldwide is canola seeds. Due to its high glucosinolate concentration and the harmful consequences of its high erucic acid level, the oil was mostly utilized as a lubricant [91]. Edible oils with high concentrations of erucic acid and glucosinolate have also been proven to raise blood cholesterol [92]. Alpha-linolenic acid, a modest amount of omega-3 polyunsaturated fatty acids, and phytosterols (approximately, 0.9% by weight) are all present in canola oil, which also has a low percentage of saturated fat (7%) [93,94]. The fatty acid profile of brassica rapeseeds contains less than 1.9% erucic acid and their solid constituents have <30 micromoles of any combination of 4-pentenyl glucosinolate, 3-butenyl glucosinolate, 2-hydroxy- 4-pentenyl glucosinolate, and 2-hydroxy-3-butenyl glucosinolate [95].

### 2.11. Rice Bran

Rice is a major global cereal crop of the family Oryzeae. Rice is a dietary staple food for about half of the world’s population [96]. The by-products of rice bran include rough rice (4.9–7.8%) and polishing (2.1–2.9%). The outer layer of the rice kernel is rice bran, which mainly consists of the pericarp, sub-aleurone layer, aleurone, and germ [97]. Rice bran γ oryzanol, tocopherols, and tocotrienols, contain appreciable quantities of antioxidants, which have been reported in a study. Rice bran is a potential source of edible oil. It consists of 14.7–21% oil, depending upon agri-climatic factors, variety, and degree of milling [98]. Rice bran oil has a high fatty acid profile, which contributes to its nutraceutical value, as do other phytochemicals (tocopherols, oryzanol, and tocotrienols) [99,100]. Rice bran oil is derived from the hard outer brown coat of rice known as bran. Its high smoke point of 232 °C and mild flavor make it ideal for high-temperature cooking methods like deep frying and stir-frying [101,102].

### 2.12. Peanut

The peanut is a legume crop farmed primarily for its edible seeds. Other names for the peanut include groundnut, goober, pindar, and monkey nut [103]. Due to its high oil content, it is categorized as both a grain legume and an oil crop. A vegetable oil made from peanuts is called peanut oil, commonly referred to as groundnut oil or arachis oil. When prepared using roasted peanuts, the oil has a greater peanut flavor and scent compared to the ordinarily mild or neutral flavor [104]. Vitamin E, an antioxidant with various advantages in preventing chronic illness, is abundant in peanut oil. The concentration of total phenolic compounds found in peanut oils is in the range of 0.98 mg to 7.36 mg/100 g of gallic acid equivalent (GAE). Indian raw peanut kernels were reported to contain the following ingredients: 48.1% fat, 26% protein, 5.4% moisture, 18.01% carbohydrate, and 2% ash. [105]. The average antioxidant activity in peanut oils is 0.81 mg/100 g and the average inhibition percentage is 18%. Eight different fatty acids make up the majority of the triglycerides in peanut oil. Oleic acid (monounsaturated, C18:1) and linoleic acid (polyunsaturated, C18:2) make up around 81% of these fatty acids [106]. The antioxidant activity in peanut seed is attributed to the vitamin E in the oil or the ferulic acid, chlorogenic acid, caffeic acid, and coumaric acid. Furthermore, it also contains a range of antioxidative phytochemicals including several phenolic acids, flavonoids, and stilbenes (e.g., resveratrol), which have health-benefiting effects through apparent anti-inflammatory, antimicrobial, and anticancer properties [107].

### 2.13. Soybean

Soybean is a key leguminous crop is Merrill. For both human and animal use, soybean seed is a crucial crop economically since it offers cheap sources of protein and oil [108]. Isoflavones, a kind of phytoestrogen found in soybeans, are present in significant concentrations; the major isoflavones are daidzein and genistein which are present in concentrations of 3.0 and 2.0mg per gram, respectively. These isoflavones can help increase glucose tolerance, while also protecting against cancer, hyperlipidemia, and cardiovascular disease (CVD) [109]. They also lower cholesterol levels in some diabetics and hyperlipidemic people [110]. Additional phenolic chemicals, proteins, volatile compounds, tocopherols, sterols, organic acids, sugars, and fatty acids are also present in soybeans [111]. The exceptions to this rule include *octanal, (E)-2-hexenal, 2-n-pentylfuran, limonene, 1-hexanol, heptanal, 3-octen-2-one, (E, Z)-2,6-nonadienal, (E)-2-octenal, (E)-2-nonenal,* and *(E, E)-2,4-nonadiena.* The soybean oils have a free radical scavenging capability of approximately 163 g/mL [112].

### 2.14. Sesame

Sesame, an herbaceous annual plant of the family Pedaliaceae and order tubiflorae, is grown for its edible seed, oil, and flavor. B vitamins, healthy fats, protein, fiber, minerals, antioxidants, and other advantageous plant elements may all be found in medium amounts in sesame seeds. Sesame plants yield seeds that are high in fiber, protein, and good fats [113]. Due to its high level of oxidation and rancidity resistance, it is sometimes referred to as the “Queen of Oilseeds” in popular culture. Sesamin, sesamolin, and tocopherol homologs, which are natural antioxidants, make up about 50–60% of the high-quality oil found in sesame seeds [114]. Phytosterols, vital minerals, polyunsaturated fatty acids, vitamins, tocopherols, and a special family of lignans, like sesamin and sesamolin, are among the seed’s bioactive components [115]. Tocopherols, phytosterols, and lignans, which are phenylpropanoid chemicals, operate as a protective mechanism against reactive oxygen species by avoiding oxidative rancidity to improve the keeping quality of the oil [116]. Sesame seeds are a major source of calcium, phosphorus, magnesium, iron, manganese, zinc, and copper, and are high in protein, dietary fiber, all eight essential minerals, and vitamin B1. Many of sesame seeds’ health-promoting properties have been linked to a class of substances known as lignans (sesamin, sesamolin, sesaminol, and sesamolinol). Sesame seed oil is known to contain lignan aglycones and lignan glucosides [117]. The oil in sesame seeds is abundant and includes significant levels of unsaturated fatty acids (81–89%), oleic acid (34–42%), palmitic acid (9–12%), linoleic acid (38–48%), and stearic acid (5.2–11%), with just traces of linolenic acid [118]. The seeds are a great source of bioactive substances, such as short-chain peptides, phytosterols, phytates, PUFA, and phenolics as well as antioxidants. Sesame cake is a fantastic source of minerals, carbohydrates, and protein [119,120].

### 2.15. Corn

*Zea mays* is the scientific name for corn. “Maize” was the Native American word for this crop. The pericarp (seed coat), endosperm, and embryo are the three crucial components of the maize kernel [121]. The pericarp guards against bacterial and fungal assault on the confined endosperm and embryo. At a level of 62%, starch makes up the majority of the kernel. The maize kernel also contains water (15%), oil (4%), protein (3.2%), and fiber (19%) [122]. Triacylglycerols make up 99% of maize oil, with polyunsaturated fatty acids making up 59%; monounsaturated fatty acids make up 24% and saturated fatty acids make up 13%. With a small quantity of linolenic acid (C18:3n-3), the PUFA largely consists of linoleic acid (n-6), giving it an n-6/n-3 ratio of 83 [123]. Due to the presence of heart-healthy compounds like vitamin E, linoleic acid, and phytosterols [124], maize oil has the potential to lower the risk of heart disease. The strong antioxidant vitamin E may protect your heart and blood vessels from oxidative damage brought on by too many free radicals [125].

### 2.16. Palm

Palm oil is a type of edible vegetable oil, in which all fats are extracted from the mesocarp or reddish pulp of the palm [126]. Palm oil is made up of fatty acids that have been esterified with glycerol. Saturated fatty acids, particularly palmitic acid and 16-carbon saturated fatty acid are particularly abundant in palm oil [127]. Palm oil is a prominent source of tocotrienol, a member of the vitamin E family. Palm oils are often preferred by food manufacturers because they are easier to maintain in terms of flavor and consistency in processed foods. In palm oil, 43% of the fat is saturated, comprising 4.8% stearic acid (C18:0), 5% myristic acid (C14:0), and traces of the other acids [128]. About 41% of the fatty acids are oleic acid and 9.9% are polyunsaturated linoleic acid. About 39.6% of the fatty acids are unsaturated, including 1% each of polyunsaturated linoleic acid, linolenic acid (C18:3), and oleic acid [129]. Tocopherols, carotenoids, and phenolic compounds are among the useful phytochemicals found in palm oil, which also has a balanced fatty acid content. These phytochemicals in palm oil protect against oxidative stress by acting as antioxidants [130].

### 2.17. Berry

Berry is an indehiscent fruit, the entire ovary wall ripens into a relatively soft pericarp, and the seeds are implanted in the ovary [131]. Cold pressing is used to extract berry carrier oils from the berry’s seed [132]. Numerous berries, such as the seeds of strawberries (*Fragaria x. ananassa*), blackberries (*Rubius fructicosus*), blueberries (*Vaccinium corymbosum*), raspberries (red and black), blackcurrants (*Ribes nigrum*), cranberries (*Vaccinium macrocarpon*), açai berries (*Euterpe oleracea*), seabuckthorn, and raspberry seeds all contain oils [131]. The berry seed oils were found to contain notable levels of several fatty acids, including palmitic acid (C16:0), stearic acid, oleic acid, linoleic acid (ω-6), and α-linolenic acid (ω-3). These oils also exhibited a favorable ratio of ω-6 to ω-3 fatty acids, ranging from 1.49 to 3.86, with significant contributions from palmitic, stearic, oleic, and α-linolenic acids. [133]. Juniper berry oil was found to have high concentrations of α-pinene (ranging from 53.6% to 62.3%), β-myrcene (ranging from 6.5% to 6.9%), and germacrene D (ranging from 4.5% to 6.1%) [134].

### 2.18. Olive Oil

The olive tree’s fruit is used to make olive oil (*Olea europaea* L.) [135]. The three primary components are cellulose (30–35%), lignin (21–24.9%), and hemicellulose (20.8–27.64%). Triacylglycerols (more than 97%) make up the majority of its composition, with a few minor components (hydrocarbons like squalene, phytosterols like β-sitosterol and -5-avenasterol, α-tocopherol, phenolic compounds, aliphatic alcohols, diterpenic and triterpenic alcohols, carotenoids, and chlorophylls). The main fatty acids found in olives are palmitoleic (C16:1), stearic, oleic, palmitic, and linolenic acids. There are trace levels of gadoleic (C20:1), margaric (C17:0), and myristic acids [136]. The primary phenolic compounds found in olive oil include the glycoside oleuropein, along with hydroxytyrosol (3,4-dihydroxyphenyl ethanol) and tyrosol. Oleuropein, a prominent phenolic compound in olive oil, is found in the concentration range of 100 to 1000 mg/kg. It exhibits robust antioxidant and anti-inflammatory attributes. Hydroxytyrosol, another essential phenolic compound, is typically found at levels ranging from 1 to 100 mg/kg. It is well-known for its potent antioxidant properties and its capacity to support heart health. Tyrosol, akin to hydroxytyrosol, is often present in similar concentrations and contributes to the oil’s overall antioxidant potential [135]. Olive oil contains caffeic acid (2 to 20 mg/kg) and p-coumaric acid (1 to 10 mg/kg), both possessing antioxidant and anti-inflammatory attributes. Luteolin, a flavonoid phenolic compound found in olive oil albeit at lower concentrations (1 to 10 mg/kg), also exhibits antioxidant and anti-inflammatory benefits. These diverse phenolic compounds collectively contribute to the health-promoting properties of olive oil. These major phenolic constituent has been reported to have the potential to reduce the risk of coronary heart disease and atherosclerosis [137].

### 2.19. Melon Seeds

Melon seeds (*Cucumis melo* L) are a good source of vitamin B3, B2, folate, zinc, magnesium, potassium, manganese, calcium, iron, and copper as well as other minerals and nutrients [138]. They make a great plant-based protein source. Melon seeds include 4.5% moisture, 25.0% crude protein, 2.4% ash, 25.0% crude fat, 23.3% crude fiber, and 19.8% carbohydrates. Sterols, tocopherol, and phospholipids were among the lipid components that were identified through analysis [139]. Oleic acid made up 24.8–25.6% of the fatty acids in the seeds, and linoleic acid made up 51.1–58.5% [140]. Other than these, the two primary triglycerides in the seeds were oleoyl dilinolein (OLL), which made up 31–34.0%, and trilinolein (LLL), which made up 31.3–32.2% [140]. Hydroxycinnamic acids (also known as caffeic, sinapic, β-coumaric, and ferulic acids), which have a low molecular weight (LMW), are important phenylpropanoids. They often have connections to the polysaccharides found in cell walls [141].

### 2.20. Pomegranate

Pomegranate seeds are a rich source of many vitamins and nutrients since they come from pomegranates, which are fruit-bearing deciduous shrubs or small trees in the family Lythraceae [142]. Pomegranate is a powerful antioxidant. This fruit is rich in flavonoids, anthocyanins, punicic acid, ellagitannins, alkaloids, fructose, sucrose, glucose, and other compounds, and has antiatherogenic, antihypertensive, and anti-inflammatory properties. The fatty acids, fiber, and antioxidants included in pomegranate seeds may be beneficial to your health. They are also a good source of vitamin E and magnesium [143]. After the extraction of the juice from the pomegranate’s seed, what is left behind as solid waste includes a variety of bioactive and nutritive substances, including fatty acids, flavonoids (such as anthocyanins), hydrolysable tannins (such as punicalagin and ellagic acid), and flavonoids (e.g., quercetin and kaempferol) [144]. Pomegranates have high manganese, phosphorus, potassium, and zinc mineral content. Iron and calcium are also present in trace amounts. The daily value for vitamin K is 16%, while for vitamin C it is 12% [145].

### 2.21. Mustard Seeds

Mustard seeds (*Brassica nigra*) are tiny spherical seeds containing 30% or more oil. White mustard, brown mustard, and black mustard (*Brassica nigra*) are the types of mustard. Around 12.9% of mustard oil is made up of saturated fat, 21% of it is made up of polyunsaturated fats, and 60% of it is made up of monounsaturated fatty acids, including 41% erucic acid, 11.8% oleic acid, 5.7% alpha-linolenic acid, and 16% omega-6 linoleic acid (ω-6 linoleic acid) [146]. According to the black mustard seed’s fatty acid composition, oleic (22.96%) is the most abundant fatty acid, after the linoleic (6.63%) and linolenic (3.22%) fatty acids [147]. Erucic acid (29.81%) and linolenic acid (49%) are the two main fatty acids in mustard oil. Erucic acid (26.5–36%) dominates the high-fat content of mustard seeds, which ranges from 23% to 47% [148]. The primary acids found in mustard seed oil are erucic, arachidic, alpha-linolenic, oleic, and palmitic. Inflammation and discomfort may be lessened with mustard oil, cancer cell development may be slowed, microbial growth may be inhibited, and mustard oil is good for hair and skin health. Mustard oil contains glucosinolates like sinigrin and gluconasturtiin. When crushed, these compounds transform into isothiocyanates and thiocyanates, lending mustard oil its sharp flavor and potential health benefits. Mustard oil’s characteristics vary due to factors like plant type and processing methods, making it a common ingredient in South Asian and Mediterranean cuisines, but it should be used in moderation [149].

### 2.22. Wheat Germ

Wheat germ oil is extracted from the germ portion of wheat grain. Bran, germ, and endosperm are the three components that make up the edible portion of the wheat kernel. The germ portion of the kernel contains 27% of the total lipids in the grains [150]. The composition of the germ lipid concentration is about 82% triglycerides, which are non-polar lipids, 16% phospholipids, and 2% glycolipids. The most commonly used conventional technique for the production of the oil is the prepress solvent extraction technique. Wheat germ oil is associated with numerous health benefits and is a highly nutritious oil [151]. Wheat germ oil contains a high amount of vitamin B and contains 1.4 g per kg of phosphorus [152]. It is high in healthful fats because of its small amount of saturated fatty acids, as well as its plant proteins, fibers, and fat-soluble vitamins such as A, D, E, and K. It exhibits high antioxidant, antimicrobial, and anti-inflammatory properties [153]. The non-polar lipids in wheat germ oil are mostly composed of unsaturated fatty acids. The most dominant fatty acid is found to be linoleic acid 56%, then palmitic acid at 16% followed by oleic acid at 13%, stearic acid at 0.8%, and linolenic acid at 8%. Wheat germ oil also contains a minute quantity of alpha-tocopherols (vitamin E) and phytosterols, such as campesterol and sitosterol [154,155].

### 2.23. Okra Seeds

The most frequently cultivated and consumed specie from the Malvaceae family is okra (*Abelmoschus esculentus*). Okra seeds contain approximately 20–40% oil [156]. It is a wholesome vegetable grown in tropical, subtropical, and warm temperate climates across the globe [157]. Okra is a cost-effective source of protein, carbohydrates, vitamins, minerals, dietary fiber, and other phytonutrients that have positive effects on the body. The substantial amount of edible oil found in okra seeds is high in mainly two types of unsaturated fatty acids, which are C18:1 and C18:2 [30]. Okra seeds contain edible oil and the leftover meal from oil extraction is incredibly protein rich. Okra seeds grown in Greece are said to contain between 15.9 and 20.7% oil. Linoleic acid makes up the majority of the okra seed oil (up to 47.4%) [158].

### 2.24. Avocado Seed

Avocado seed contains bioactive elements such as phenolics, flavonoids, carotenoids, vitamin C, and vitamin E [159]. The avocado seed contains phytochemicals like flavonoids, tannins, saponins, phenolics, antioxidants, oxalates, phytates, and alkaloids [160]. Additional polyphenolic compounds within avocado seeds include 3-O-caffeoylquinic acid, 3-O-p-coumaroylquinic acid, procyanidin trimer A(I), procyanidin trimer A(II), catechin, and epicatechin gallate. Other research suggests the presence of tannins, saponins, and flavonoids as constituents of the polyphenols in avocado seeds [161,162]. Avocado seed contains phytochemicals like flavonoids, tannins, saponins, phenolic acids, and alkaloids [163].

### 2.25. Nigella Seeds

Nigella seeds, also called black seeds, belong to the family Ranunculaceae. Black seed oil contains 58% of linoleic acid which makes it a healthy and nutritious oil. In addition, it also contains important minerals, such as calcium, sodium, copper, magnesium, and iron [164]. Black seed oil shows high antimicrobial properties as it contains 28% cymene, 39% thymoquinone, and 5.9% longifolene. The abundant amino acids present in black seed oil include aspartic acid, glycine acid, arginine, and glutamic acid [165]. The black seed oil contains high amounts of linolenic and oleic acids, which is about 58% and 25%, respectively. It also contains 12% of palmitic acid, 2.5% of stearic acid, and 2% of eicosadienoic acid. Black seeds include a variety of vitamins and minerals, e.g., calcium, iron, zinc, and copper, as well as saponin and alpha-hederine in trace levels [166]. The predominant volatile compounds present in black seeds consists of p-cymene (36.35%), methyl linoleate (1.33%), thymoquinone (29.77%), carvacrol (2.85%), β-pinene (2.41%), limonene (1.64%), α-thujene (12.40%), and sabinene (1.18%). The combined proportional contribution of these compounds to the overall volatile oil content reached 87.93% [167]. The thymoquinone is the most prevalent component present in black seed oil, and causes the majority of the pharmacological actions. The thymoquinone present in black seed oil possessed high antioxidant properties, anticancer properties, anti-inflammatory properties, and antimicrobial properties. Anticonvulsant, antioxidant, anti-inflammatory, anti-cancer, antibacterial, and antifungal properties are all possessed by thymoquinone [168].

### 2.26. Cotton Seed

Cotton seed is an economic crop because of its oil and other goods. After being hydrogenated, cottonseed oil is used to make shortenings and margarine in addition to salad and cooking oils. The fatty acid composition of cottonseed oil is composed of 55% polyunsaturated fat, 26% monounsaturated fat, and 26% saturated fat [169]. Of the total fatty acids, 3% are stearic acid, 22% are palmitic acid, and 54% are linoleic acid, followed by 52% of linoleic acid, 25% of palmitic acid, and 16% of oleic acid, along with trace quantities of myristic and stearic acid. It is known as anti-inflammatory vegetable oil, and exposure to light, air, and high heat causes it to rapidly oxidize. Cotton seed oil contains high amounts of omega-6 and omega-3 fatty acids [170]. Gossypol is a phenolic compound synthesized by pigment glands in cotton plants. There are two observed forms of gossypol in cotton: the free form and the bound form. The bound form is created through covalent bonding between gossypol and the epsilon-amino groups found in lysine and arginine. Elevated concentrations of free gossypol may lead to acute clinical symptoms of gossypol poisoning, which encompass respiratory difficulties, reduced body weight gain, loss of appetite, weakness, lethargy, and eventual death after several days [5]. One of the most prevalent toxic effects of gossypol is its adverse impact on both male and female reproductive functions. Proper purification of cottonseed oil is essential to eliminate gossypol from the oil [171].

### 2.27. Sea Buckthorn Seeds

*Hippophae rhamnoides* L., commonly referred to as sea buckthorn and belonging to the Elaeagnaceae family, boasts a rich mineral composition including calcium (Ca), phosphorus (P), iron (Fe), and potassium (K). Notably, sea buckthorn stands out with its elevated vitamin C content compared to other fruits, containing approximately 360 to 2500 mg/100 g of vitamin C. Abundant in vitamins, the plant offers a generous supply of vitamin B, particularly B1 (thiamine) and B2 (riboflavin), as well as vitamin E, A, and K [172]. The berries of sea buckthorn are a valuable source of carotenoids such as ß-carotene, lycopene, lutein, and zeaxanthin. In terms of carbohydrates, glucose, fructose, and xylose are the predominant components. Proteins are diverse across various parts of the plant. Renowned for its wide range of health benefits, sea buckthorn showcases antioxidant, cardioprotective, antiatherogenic, antidiabetic, hepatoprotective, anticarcinogenic, immunomodulating, antiviral, antibacterial, anti-inflammatory, and vasodilating properties [173].

### 2.28. Chaenomeles ssp. Seeds

The Chaenomeles shrubs, commonly known as quince, belong to the Rosaceae family’s Maloideae subfamily. Quince seeds contain fourteen phenolic acids, with caffeic acid, protocatechuic acid, gallic acid, p-hydroxybenzoic acid, p-coumaric acid, syringic acid, and vanillic acid being the predominant compounds [174]. These seeds possess potent antioxidant properties due to their high content of phenolic acids, isoflavonoids, catechins, anthocyanidins, phytoalexins, and tannins. Quince fruits offer antibacterial, anti-inflammatory, antiproliferative, antidiabetic, antihyperlipidaemic, antihyperglycemic, immunomodulatory, and antioxidant benefits. Valuable amino acids and proteins are found in quince seeds, which display actions such as antioxidant, anticancer, anti-hepatitis, antimicrobial, immunoregulatory, anti-influenza virusl, and anti-Parkinson’s effects [175].

### 2.29. Hazelnut Oils

Hazelnuts are tree nuts with thick shells, and their oils can be consumed either in their raw form, preferably after roasting the seeds, or after being refined. Hazelnuts, scientifically known as *Corylus* sp. L., belong to the birch family, Betulaceae. The oil extracted from hazelnuts is characterized by high levels of oleic acid (68.8–78.6%) and linoleic acid (14.2–23.3%), while containing lower quantities of palmitic acid (4.5–5.9%), stearic acid (0.5–2.8%), and linolenic acid (0.1–0.2%). Oleic acid stands out as the most abundant fatty acid, with oleic acid and linoleic acid together constituting over 90% of the fatty acid composition in all hazelnuts [176].

## 3. Extraction Techniques

Many methods have been used to extract oil from seeds and other edible plant parts. In recent decades, non-conventional extraction techniques have been described, including those listed below and summarized in Table 1.

### 3.1. Microwave-Assisted Extraction (MAE)

Microwave pre-treatment of oil seeds provides an interesting alternative to conventional extraction techniques. Studies have concluded that microwave-assisted extraction not only increases the net yield of oil extraction, but it also improves the overall phytochemistry and functional properties of the extracted oil as compared to the oils extracted using conventional methods. Microwave extraction reduces extraction time and the volume of solvent required. Moreover, oils extracted through microwave roasting present improved oxidative stability, hence improved shelf-life. In additon, this technique enhances the antioxidant abilities and phenolic profile of the extracted oils [177].

In microwave-assisted extraction, the sample is subjected to non-ionizing radiations, with frequencies in the range of 300 MHz and 300 GHz. These electromagnetic radiations cause ionic conduction, forcing the molecules to align to the electric field of radiation. This phenomenon generates heat and evaporates the water content present in the sample, increasing the pressure inside the sample cells. The tissue cells of the sample burst under high pressure, releasing the bioactive components to be extracted. Microwave-assisted extraction is also known as solvent-free microwave extraction (SFME) as no solvent is involved. The efficiency of the method depends upon the frequency of the microwaves, time duration, and initial water content in the sample matrix [178].

### 3.2. Pressurized Liquid Extraction (PLE)

Pressurized liquid extraction, also known as accelerated solvent extraction (ASE) is yet another emerging extraction technique. PLE extracts bioactive components by using a combination of both water and a solvent at high temperatures and pressure. Oils extracted through PLE are enriched in phenolic acids, polymethoxylated flavones, terpenoids, polyphenols, etc. Given the rich phytochemical profile, the extracts possess high antioxidative, radical scavenging activity.

PLE, a green extraction technique, is one of the most time-efficient and economic extraction techniques. High pressure allows the liquids to be subjected to temperatures higher than their atmospheric boiling points without being boiled. This arrangement results in an increase in the bioactive components’ solubility and diffusion into the solvent. On the other hand, it also significantly decreases the surface tension and viscosity of the solvent. All of these factors allow maximum extraction of the components of interest after complete drainage of the sample matrix. PLE is performed in a dynamic setup, in which the solvent is delivered at a constant flow rate, while the static mode of operation involves solvent replacement between one or more cycles in a predetermined time [179].

### 3.3. Cold-Pressed Extraction (CPE)

Cold-pressed extraction is one of the extraction techniques that are performed under low temperatures. This extraction technique does not require a solvent or any additional sample processing. Extracts obtained using cold pressing have a dense phytochemical profile due to low temperature and also because the whole process is completed at low temperature and no solvent or chemical of any nature is required. Additionally, oils extracted with this technique have higher total tocopherol, oxidative stability, and antioxidant properties as compared to those obtained from solvent extraction. Similarly, this technique ensures high quality and high concentration of bioactive compounds in the extracted oils. Cold-press extraction is a cost-effective, green, and safe extraction technique, providing pure and nutritionally rich oils but the comparatively less net yield is a drawback [180].

The main objectives of this technique are efficient use of energy, minimum to no impact on the environment, as well as minimal loss of bioactive compounds. The process includes feeding the oil seeds into a hopper that drops them at a constant rate into a mechanical screw, which acts as a worm conveyer, fitted inside a barrel with slotted walls. The screw puts the oil seeds under very high pressure, extracting oil that passes through the slots inside the walls of the barrel and is collected underneath. The dry material that is left, termed as cake, is passed out separately [181].

### 3.4. Ultrasound-Assisted Extraction (UAE)

Ultrasound-assisted extraction has become a widely preferred method of extraction due to its low working temperature, low solvent requirement, and reduced processing time. Studies have concluded that oils extracted through UAE have no significant structural and functional change in fatty acids. Additionally, it is also observed that such oils have higher concentrations of polyunsaturated and monounsaturated fatty acids, as well as reduced concentrations of saturated fatty acids [182]. Reduced time duration of the extraction process enhances the stability of oils. Similarly, UAE may also enhance the antimicrobial properties of the extracted oils. The functional parameters for ultrasonication vary from a few tens of watts to hundreds of watts, usually 20–40 kHz, and varied time duration.

The ultrasonic technique is considered to be a green technology due to its lower cost, reduced operation cost, higher yield, and lesser energy consumption. Ultrasonic radiations have enhanced efficiency due to cell wall disruption and mass transfer. Ultrasound radiation produces cavitation bubbles that lead to the breakage of cell walls and water channels. These physical changes increase the contact area of bioactive compounds and solvents, increasing the mass transfer of phytochemicals. Bioactive compounds may come out via diffusion or dissolution [183].

### 3.5. Supercritical Fluid Extraction (SFE)

The primary component of supercritical fluid extraction is a supercritical fluid. The triple point of a matter is when they co-exist in all three forms, i.e., solid, liquid, and gas at a specific temperature and pressure. When the temperature and pressure increase, the phase difference between solid, liquid, and gas diminish. This event is known as the critical point, at which the matter has the solubility and density of a liquid and behaves like a gas. The critical point for carbon dioxide exists at a critical temperature of 31 °C and critical pressure of 74 bar [184].

Extraction in this technique occurs at two difference stages i.e., diffusion and dissolution. The supercritical CO_2_ behaves like a gas and can readily infiltrate into the solid matrix. It is capable of penetrating small pores of the sample matrix. Similarly, high solubility and density of the supercritical CO_2_ can widely dissolve the extract and bring it from the solid matrix to the outer layer, followed by taking it to the solution. CO_2_ is s preferable solvent in this technique because it is widely available in the environment, can be recycled, leaves no traces inside the sample, and has low critical temperature and pressure [185,186].

### 3.6. Enzyme-Assisted Extraction (EAE)

Enzyme-assisted extraction method involves the use of certain enzymes to assist bioactive compounds’ extraction. It is a green and preferred method of extraction without using denaturing conditions, such as solvents, high temperature, light, etc. Its advantages include catalytic efficiency, mild aqueous conditions, high specificity, biological activities, high yields, low energy consumption, and cost [187]. The most commonly used enzymes include pectinases, amylases, proteases, and cellulases. Studies have concluded that extracts obtained by using pectinase enzyme result in promoted immunostimulatory activities. Moreover, it is also reported that extracts obtained employing enzyme-assisted extraction possess more than twice the antioxidant activity than those of extracts obtained through conventional methods [188].

This technique involves the catalytic metabolism of polymers in the cell wall, such as hemicellulose and cellulose. These enzymes break cell walls releasing the bioactive compounds which are bonded with the cell wall either through hydrogen bonding or enclosed inside hydrophobic pockets with the cell wall. Hence, these enzymes free the bioactive components and are easy to be further extracted. Additionally, the high specificity of the enzymes being used for extraction enables the development of tailor-made extraction formulation [187].

### 3.7. Pulsed Electric Field-Assisted Extraction (PEA)

Pulsed electric field-assisted extraction is a non-thermal technique that uses electric fields for the extraction of bioactive compounds. Electric fields either increase the solubility of the phytochemicals or the permeability of the cell membrane, improving the extraction. This technique increases the release of polysaccharides and proteins from the sample matrix. Similarly, it also enriches the extract with polyphenols, carotenoids, and other pigments. The pulsed electric field also enhances the antioxidant properties of the oil, thereby increasing the stability and shelf-life of the product [188]. The PEF technique is carried out in batch and continuous systems in two phases with an electric field strength of 100 to 300 V/cm and 20 to 80 V/cm, respectively. Electric field strength and time of application depend upon the type of cell and extractant. There are two proposed mechanisms: the electric field imparts structural changes to the bioactive compounds, increasing their solubility; on the other hand, the electric field increases cellular permeability by creating hydrophilic pores in the cell membrane, resulting in the release of phytochemicals. This structural change may either be temporary or permanent depending upon the energy of the electric field applied [189].

**Table 1:** Extraction methods of fixed oil from plant seeds.

Seed Name	Scientific Name	Oil Content	Extraction Method	Conditions	Efficiency and Yield (%)	Advantages	Disadvantages	References
Flax seed	*Linum usitatissimum*	35–45%	Microwave-assisted extraction (MAE)	50% power level heating time 10 s, temperature ranging from 55 to 60 °C	75%	Higher yield, shorter extraction time, high purity of final product, green technology	High maintenance cost	[190]
Chia Seed	*Salvia hispanica*	25–40%	Pressurized liquid extraction (PLE)	---	83%	Highly effective, authenticity, high accuracy	Expensive It needs experienced operator	[191]
Tomato seed	*Solanum lycopersicum*	25%	Cold-pressed extraction (CPE)	The cold pressing set by a 10-mm exit die, and 40 rpm of screw presses at 40 °C	12.80–9.66%	Simpler, lower operation cost, natural compounds are preserved in cold pressed oils	Oil yield is not high	[192]
Clove seed	*Syzygium aromaticum*	15–20%	Microwave-assisted extraction (MAE)	Extraction tim10 min, power 150 W and temperature 50 to 60 °C	up to 41.8%	Less solvent use, higher extraction yield	Higher cost of instrument	[75]
Soya been seed	*Glycine max*	18–22%	Ultrasound-assisted extraction (UAE)	Power (60, 80, and 100%), sonication time (20, 40, and 60 min), and solvent volume (75, 100, and 125 mL)	48–84%	Fast, high extraction yield, less solvent use, environmentally friendly	Costly method	[193]
Avocado	*Persea americana*	Up to 30% oil	Cold-pressed extraction (CPE)	screw presses at low temperatures (<50 °C)	60–80%	Fast, less heat and solvent use	Lower productivity	[194]
Pomegranate seeds	*Punica granatum*	7.6–16.2%	Ultrasound-assisted extraction (UAE)	extraction temperature, 20 C; solvent/solid ratio, 20/1; amplitude level, 60%; pulse duration/pulse interval ratio, 5/15.	59.8%	Shorter extraction times, low energy demands and high extraction rate	Expensive	[195]
Okra seed	*Abelmoschus esculentus*	15.9–20.7%	Cold-pressed extraction (CPE)	---	15.92–17%	Less energy requirement, environmentally friendly	Oil yield is not high	[196]
Coconut	*Cocos nucifera*	63–70%	Supercritical-fluid extraction (SFE)	Pressure and temperature ranges of 20.7–34.5 MPa and 40–80 °C	80–85%	Less solvent use, greater extraction rate, low temperature	Very expensive and complex equipment	[197]
Melon seed	*Psenopsis anomala*	19.4–33.0%	Supercritical carbon dioxide (CO_2_) extraction	Pressure 44 MPa, temperature (40–50 °C), and extraction time (60–120 min)	48%	Higher extraction rate, lower environmental impact	Expensive	[198]
Tea seed	*Camellia sinensis*	30–32%	Microwave-assisted extraction (MAE)	Microwave power 440 W at 70 °C for 38 min	31.52%	Greater extraction rate within a short time, high quality oil, small amounts of solvent use	Higher capital cost	[199]
Black seed	*Nigella sativa* L.	43%	Ultrasound-assisted extraction (UAE)	ultrasound power (150, 200, 250 W), treatment time (15, 30, 45 min)	39.93%	Very efficient, fast, green techniques	Expensive method of extraction	[200]
Pumpkin seed	*Cucurbita maxima*	10.9–30.9%	Enzymatic-assisted extraction	---	36.0%	Less solvent use, shorter extraction times, and greater extraction yield	Higher cost	[201]
Berry seed	Pericarpium	11–23%	Supercritical CO_2_ extraction	Extraction pressure 350 bar, and the extraction temperature 50 °C or 80 °C and extraction time 60 min.	15%	Green extraction techniques, very fast and efficient, maintains the quality of the final product	Very expensive and complex equipment operating at elevated pressures, high power consumption	[202]
Cotton seed	*Gossypium herbaceum*	15–20%	Ultrasound-assisted extraction	Extraction time 1 h and temperature 45 °C	38.25%	Fast, efficient, and use of less solvent volumes, few instrumental requirements and less environmental impacts	Low capacity per batch, high power consumption	[203]
Coriander seed	*Coriandrum sativum*	0.1–0.36%	Ultrasound-assisted solvent extraction	sample solvent ratio of 1:13 g/mL, amplitude level of 82%, temperature of 45 °C, and extraction time of 9 min.	30.75%	Higher extraction rate, shorter extraction time, reduce process costs, more efficient heat flow, lower solvent,	Higher capital cost	[204]
Sesame seed	*Sesamum indicum*	50–60%	Pulsed electric fields-assisted extraction	pulsed electric field strength 13.3 kV/cm and duration time 10 μs	30–40%	Non-thermal equipment, fast, very efficient, high extraction rate	High capital cost	[205,206]
Sunflowers seed	*Helianthus annuus*	35–42%	Cold-pressed extraction	---	33–39%	No impurities, saves energy, fast, and completely preserves the physiologically active substances in the oil	Oil yield is not high, expensive	[207,208]
Poppy seed	*Papaver somniferum*	34.56–44.76%	Cold-pressed Extraction	---	40–45%	Reduction of processing time, low temperature	High capital cost	[28]
Mustard seed	*Brassica juncea*	30%	Cold-pressed extraction	diameter of 0.47 mm time 1 min	64.3%	Higher extraction efficient, time saver	Expensive	[209]
Olive	*Olea europaea*	20–30%	Ultrasound-assisted Extraction	Frequency 60.0 kHz, power 280 W, time 60 min	7–11%	Less extraction time and more yield at low temperatures	Expensive, lack of uniformity in ultrasound energy	[210]
Wheat Germ	*Triticum aestivum*	7–9%	Supercritical CO_2_ extraction	Pressure 300 bar, temperature 40 °C, and 8 h time	9%	Fast, efficient, environmentally friendly method, time saver	Requirement of high pressure, costly method	[211]
Rice bran	*Oryza sativa*	10–23%	Supercritical CO_2_ extraction	Extraction time 30 min, temperature 40–60 °C, pressure 30 and 40 MPa	9.2–10.2%	Green extraction, less solvent use and pure extract, low temperature use	Very expensive and complex equipment	[212]
Rape seed	*Brassica napus*	30.6–48.3%	Ultrasound-assisted extraction	Ultrasound power of 400 W, frequency 12 kHz, time 10, 20 or 30 min	80%	Higher extraction rate, shorter time, less heat and very fast extraction of bioactive compounds	Higher capital cost	[213]
Moringa seed	*Moringa oleifera*	35–45%	Microwave-assisted extraction	Microwave power ranging from 300 to 900 W microwave, time ranging from 5 to 13 min.	91–94%	Use of low solvent volumes, fast, environmentally friend less time required, more efficient	Expensive	[214]
Papaya Seed Oil	*Carica papaya* L.	29.16%	Enzyme-assisted extraction (EAE)	---	78%	Lower solvent consumption, shorter extraction times, and milder condition	Enzymes are relatively expensive and enzyme cannot break down the plant cell walls completely	[215]
Peanut Seeds	*Arachis hypogaea*	50%	Enzyme-assisted extraction (EAE)	Enzyme concentration 2.5%, temperature 40 °C, and time 18 h	80–90%	Green techniques, low solvent, fast	Costly method	[216]
Perilla seed	*Perilla frutescens* L.	35–45%	Ultrasound-assisted extraction (UAE)	250 W of ultrasonic power, 30 min of ultrasonic time, and 50 °C of ultrasonic temperature	81.74%	Higher extraction efficiency, lesser time, and effective for heat sensitive compounds	Decline of power with time, expensive method	[217]
Corn seed	*Zea mays*	3–4%	Enzyme-assisted extraction (EAE)	---	80%	Higher extraction yield, higher quality of extract, green extraction, less use of solvent	Expensive, not feasible for some materials	[218]

## 4. Methods for Quantification and Detection

### 4.1. High-Performance Liquid Chromatography (HPLC)

Oils are rich in antioxidants and terpenoids. They possess pharmacological properties which include antiseptic, antispasmodic, anti-inflammatory, and antiallergenic properties. Quantification and determination of different components of an oil can be carried out with high-performance liquid chromatography. The quantification of components present in oils varies concerning seasonal variations and geographical regions. The precise and accurate amounts of all the components were analyzed in different concentrations with HPLC techniques [219].

HPLC can be coupled with MS/MS analysis to evaluate the oxidation reactions in oils. The oxidation mechanism in canola oil is determined by quantification of hydroperoxide positional and cis–trans isomers (HpODE). In previous studies, the HpODE is determined in the presence of sodium because it is readily dissolved in sodium acetate. Therefore, ESI is coupled with HPLC-MS, which is operated in positive ion mode for the identification of possible triglyceride oxidized products. For this purpose, a simple silica-based (NP) column is considered effective in the separation of hydroperoxide groups. Other solvents are inadequate for ESI to be used in the NP column as a mobile phase because of their reduced proton transfer potential. Methanol/2-proposal in a 1:1 volume ratio is used as an HPLC eluent to increase ionization. All isomers are detected under these optimized HPLC/MS conditions [220,221].

### 4.2. Gas Chromatography–Mass Spectrometry (GC-MS)

The most commonly employed analytical platform for volatile profiling is GC-MS. Gas chromatography is sometimes coupled with mass spectrometry and uses a capillary column. The fatty acid profile, total phenolic, and flavonoid compounds are determined through gas chromatography. The solid-phase microextraction (SPME) is used for the extraction of volatile compounds from different oils with carbowax. The samples then require a methylation process before being transferred to gas chromatography. The fatty acids are nonpolar, so they cannot be analyzed with gas chromatography, for this purpose the fatty acids are converted into the gaseous state in the form of methyl esters so they become polar and are then transferred into gas chromatography [222].

In the method described in various studies, the BPX5 column is used with an initial temperature of 40 °C, then the final temperature is raised to 200 °C, with a 5 °C increase per minute [219]. Component analysis of oils, compositions of oils, and fatty-acid profile of oil were identified and quantified by using GC analysis. Gas chromatography CP-3800 was coupled with a flame ionization detector (FID) using a split and splitless injector. Capillary columns of cyanopropyl were used with the following specifications: a DB-225ms column and a DB-23 columns (30 m × 0.35 mm) and a film-thickness of 0.25 µm. Helium gas is used as a carrier gas with a flow rate of 1 mL per min. The temperature is maintained at 60 °C to 200 °C. The standard compounds mainly used are palmitic acid, linoleic acid, α-linoleic acid, erucic acid, myristic acid, palmitic acid, and stearic acid. Separation of components was accomplished via an HPLC 5 m-column of 5% phenyl methyl siloxane solution with dimensions of 30 m × 0.35 mm with a 0.1 mm diameter. The temperature of the injector port and the detector was maintained at 150 and 250, respectively. The initial temperature of the chamber was maintained at 100 °C for 4 min, followed by a gradual increase at the rate of 5 °C per min until 130 °C was reached and then the temperature was held for 20 min. The carrier gas was helium which had a linear velocity of 1.2 mL, as reported in [223]. This technique improved the separation process, and exhibited better and faster run times, better sensitivity, and high peak capacity of samples, resulting in more reliable compound identification and quantification. 2-D Gas-chromatograms provide discrete fingerprinting of samples that facilitates the better identification, classification of specific compounds, and quantification of peaks of samples, as compared to traditional 1D GC chromatograms. In a novel analytical approach, a dual secondary column configuration of comprehensive GC × GC was combined with mass-spectrometry in parallel. A flame ionization detector was employed to quantify more than 54 fatty acid profiles accurately in a single run time. FID detection was found to be more precise than MS detection for quantification purposes. For the identification and determination of authenticity of adulteration in oils, a study analyzed multi-element isotope ratios (18O, 2H, and 13C) of linalool-linalyl acetate using enantioselective multidimensional GC–MS [224].

The adulterated oil samples analysis and the comparison of their chromatographic and spectroscopic data are being used as benchmarks to analyze whether oil samples are adulterated with an unknown source. Gas chromatography is the preferred method for separating and identifying the components of oils, a technique that has been in use for years. Typically, the two main detectors, the flame ionization detector and the mass spectral detector, have been used for performing oils analysis. This separation process involved splitless injection at 0.1 L, and then further followed up by the removal of the injection port after 55 s of injection. Infrared spectra were obtained through the use of HP-5965B with a rate of 10 scans per second. This means that one spectrum was collected per second. A second derivative wavelength is used to produce infrared reconstructed chromatograms by focusing on the range of 3100 to 2800 cm^−l^. For the mass spectra analysis, the HP-5970B was used in the scanning range of 20 and 275 D; the flow rate for this process was 1.3 spectra/s, as reported in [225]. Capillary gas chromatography (CGC) primarily serves the purpose of sterol and adulteration analysis. To conduct this analysis, the sterol fraction is extracted from the unsaponified material through thin-layer chromatography (TLC) prior to the actual CGC analysis. This intricate process, which is a component of the established methods used for monitoring edible oils, presents challenges in terms of automation and throughput, thereby restricting the daily volume of samples that can be processed [226].

### 4.3. Fourier Transform Infrared Spectroscopy (FTIR)

FTIR is utilized for the identification and quantification of functional compounds with slight modifications in the sample preparation [227]. FTIR is used for the authenticity of adulterations in various vegetable oils at low prices. The oxidation process of oils yields the mechanism of free radical generations, mainly hydroperoxides and secondary oxidation products like aldehydes, ketones, etc. FTIR is used for the measurement of the quantity of these primary and secondary metabolites of oxidation, which tells the degree of the oxidation process [228]. The qualitative determination of organic compounds is accomplished by employing FTIR-spectroscopy analysis. This analysis can determine the compounds by the difference in the intensity and frequency of the edible oils. It can also be used to determine the authenticity of adulteration of oils with variable vegetable oils and fats. The FTIR spectrum is employed for the specific oil, observance of band can be conducted at 3009 per cm. The samples are heated and exposed to UV radiations at different temperatures. The spectral bands are in the range of 3005–2800 and 1775 cm^−1^. The volatile compounds determination, the fatty acids profile, and tocopherols are analyzed by using an FTIR spectrophotometer [229].

### 4.4. Gas Chromatography-Infrared Spectroscopy (GC-FTIR)

Identification and quantification of oil chemical composition and components can be realized with gas chromatography coupled with different detectors. For the separation of different fractional components of oils, gas chromatography coupled with mass spectroscopy is extensively used. As reported in research from the literature, oils contain a complex mixture of various compounds of different molecular weights (MW), including sesquiterpenes, which are oxygenated compounds of 15 carbon atoms, and monoterpenes, which are volatile hydrocarbons. To further investigate the components of oils, gas chromatography combined with a flame ionization detector was used in [230]. In the work reported in different studies, the separation of volatile compounds and heavy terpenoids was conducted at different temperatures, starting at 4 °C and temperature was increased to 200 °C. GC-FTIR mixed isolation spectra were obtained by using gas chromatography coupled with mixed isolation/Fourier transform infrared spectrometry. The equipment is assembled and conjugated using gas chromatography with mixed isolation cryogenic apparatus and FTIR spectrometry attached with different computers for data collection and control of the instruments. Helium gas mixed with argon gas was used as carrier gas, as reported in [231]. In another study, gas chromatography was coupled with columns with specific dimensions. The results of this analysis were used to determine the chemical components and composition of oil and their impact on biofilm formation by *Escherichia coli*. The injector temperature was 250 °C and the detector temperature was set as 300 °C. Helium gas was used as a carrier gas at a flow rate of 1.3 mL per min. The column’s initial temperature was maintained at 150 °C for 2 min and increased up to 260 °C at an increased rate of 5 °C per min, as reported in [232,233].

### 4.5. Atomic Fluorescence Spectroscopy (AFS)

Atomic fluorescence spectroscopy is a time-saving, rapid, reliable, and efficient analytical technique for the identification and extraction of different minerals and elements, such as mercury and arsenic, from oil samples. In a work conducted by Valasqus et al., 2020, the determination of different elements was accomplished via AFS coupled with the vapor generation method and it involves emulsion breaking and ultrasonic energy to promote emulsification. Variables obtained via the multivariate approach were optimized to achieve the best analytical characteristics for the method. Arsenic concentrations in the samples ranged from <LQ − 1.4 µg L^−1^ and selenium concentration was in the range of 3.4–15 µg L^−1^. All samples analyzed had mercury concentrations below the limit of quantification [234,235].

### 4.6. Electron Microscopy (EM)

According to a past study, the chemical composition and morphology of oils were determined with microscope techniques through the determination of oil components, as their proportion and types of terpenes were determined, and the activity of their oil components and their effect on other constituents was investigated. The study aimed at investigating the effect and influence of biologically effective UV-B radiation on the oil quantity and quality as well as specialized oil glands of field-grown basil (*Ocimum sanctum* L.). The plants were exposed to UV-B radiation, which was compared to the ambient radiation level of 1.8 kJ m^−2^ per day. To examine the surface anatomy and oil profile structure of the oil components and the morphology of the basil leaves, scanning electron microscopy was used. Similar morphology was shown on both the adaxial and the abaxial surfaces of the basil [236]. To prepare samples for scanning electron microscopy (SEM), fresh leaf sections, from the middle part without leaves, measuring about 5 mm were prepared. Several treatments were used for both control samples and ultra-violet (UV-B) treatments. The samples were dipped into the 2% solution of glutaraldehyde containing 0.1 M buffer of sodium phosphate solution at room temperature for 12 h. After fixation, washing of samples was conducted overnight three times at four temperatures with 25 Mm buffer solution containing sodium phosphate at 6.8 pH, followed by dehydration using a series of 15 min steps in different percentages of ethanol, and then stored at −20 °C until needed. Before SEM analysis, the samples were post-fixed in a 2% solution of osmium tetroxide, followed by coating with gold palladium. The coated specimens were mounted on aluminum stubs and morphological analysis was accomplished using an LEO stereoscan SEM [237].

## 5. Essential Components in Oils

Essential components in fixed oils are tabulated in Table 2.

### 5.1. Papaya Seed Oil

The fruit of the papaya is an exceptionally high-yielding crop that emerged across several tropical nations and belongs to the Caricaceae family. Stearic, oleic, linoleic, and palmitic acids were identified to be the primary fatty acids found in papaya seed oil [238]. It was discovered that papaya seed oil comprises significant quantities of benzyl isothiocyanate, which gives the oil an anticarcinogenic ability [239]. The intake of reasonable quantities of oil with a considerable oleic acid composition has been demonstrated to help increase plasma lipid status as well as stop heart attack and stroke because the oleic acid may reduce cholesterol and plasma triacylglycerol quantities without influencing plasma low-density lipoprotein (LDL) cholesterol concentrations in healthy normolipidemic individuals. This observation has been confirmed with several epidemiological analyses, research on animals in laboratories, and clinical experiments [240].

### 5.2. Flaxseed Oil

The flax plant, a perennial plant and a member of the Linaceae family, yields flaxseed as its seed [82]. Flaxseed oil, also known as linseed oil, provides excellent dietary and functional characteristics. The abundance of components, including vitamin E, dietary fibers, lignans, polyunsaturated fatty acids, and essential amino acids, make flaxseed a great alternative for achieving essential nutritional needs and maintaining optimal health. Antioxidant, anti-inflammatory, nutritious, and anticarcinogenic attributes contribute to reducing cholesterol levels, managing coronary heart diseases, and controlling hyperglycemia [61,190]. The human malaria parasite, Plasmodium falciparum, is impeded in growth by biologically active peptides isolated from flaxseed, including cyclolinopeptide A, a compound with powerful immunological and antimalarial effects [241]. It contains α-linolenic acid, which accounts for 55–60% of all its fatty acids. Protein, fat, and dietary fiber are all abundant in linseed. Linseed typically comprises moisture, ash, protein, dietary fiber, and oil. Fat, dietary fiber, protein, and carbohydrates, and soluble and insoluble fiber, magnesium, potassium, zinc, and B vitamins, are the main components of linseed. The greatest amount of omega-3 fatty acid approximately 50–60%, 73% PUFA, 18% MUFA, 9% SFA, and 55% of total fatty acids are found in linseed oil. Lignans are a good source of phytoestrogen that may help prevent breast cancer [225]. According to previous studies, linseed shows bioactive peptides that have bio-digestibility, antiproliferative, anti-Parkinson’s, antimicrobial, anticancer, antiulcer, immune-stimulating, antioxidant, and antihypertensive effects [242].

### 5.3. Eucalyptus Oil

Eucalyptus plants have drawn special consideration in numerous manufacturing industries, particularly furniture, nutraceuticals, scents, and medicines. They provide a rapidly developing source of wood along with an oil source that may be utilized to produce many different kinds of items [22]. Gum, cellulose, and wood may be produced from eucalyptus, and the eucalyptus oil that is derived from the leaves is applied substantially in medicinal products, cosmetics, and food categories [243]. Terpenes and phenolic compounds are among the most prominent phytochemicals in numerous eucalyptus species. The application of the oil of eucalyptus in personal care and beauty goods is expanding considerably. Furthermore, it has been extensively applied in conventional medical practices for managing respiratory ailments including the common cold, influenza, and congestion in the sinuses [244].

### 5.4. Olive Oil

The main element of oil extracted from olive seeds are triacylglycerols. The rest of the TGA in olive oil is composed of stearic acid, linoleic acid, palmitic acid, and palmitoleic acid. In recent research, polyphenols decreased death rates and prevented the development of cardiovascular, cancer, and neurological conditions. Polyphenolic components, such as oleuropein, hydroxytyrosol, and derived compounds, occur frequently in olive oil. They are effective antioxidants having anti-inflammatory, anti-angiogenic, and anticancer activities [245].

### 5.5. Camelina Oil

*Camelina sativa* is a plant that grows annually, having an average lifespan of 85–100 days, and is an oilseed plant and a member of the Brassicaceae family [246]. Camelina seeds are an excellent provider of vitamin E and total phenolics and also contain polyunsaturated and monounsaturated fatty acids [247]. In camelina seeds, studies have reported eleven phenolic acids, three carotenoids, nineteen fatty acids, and eight flavonoids. C-11-Eicosenoic, oleic, α-linolenic, and linoleic acids are among the four essential fatty acids [248,249]. Globulins, glutelins, and albumins, among different protein categories with various solubilities, are all found in camelina proteins [250]. Furthermore, the human body may change α-linolenic into the by-products of metabolism, such as eicosapentaenoic and docosahexaenoic acids, both of which may help lower the chances of coronary heart problems [251] and promote psychological health [252].

### 5.6. Grape Oil

Anti-inflammatory, antibacterial, cardio-protective, and anticancer effects of the oil extracted from grape seeds have been primarily identified through in vitro testing. It can additionally interact with cellular and biochemical mechanisms. The chemical constituents of grape seed oil—such as resveratrol, linolenic acid, quercetin, procyanidins, phytosterols, carotenoids, and tocopherol—have been associated with these activities [253]. In addition to its significant antioxidant capacity, neuroprotective qualities, and anticancer effects, vitamin E enhances the nutritional benefits associated with grape seed oil [254].

### 5.7. Safflower Oil

One of the world’s oldest oil crops is the safflower. Safflower oil has the maximum linoleic acid concentration of any industrial oil and is composed of stearic, linoleic, oleic, and palmitic acids [255]. Safflower oil is a great dietary supply of vitamin E, due to its high tocopherol concentration, but it has low thermostability for high-temperature uses like deep frying or lubricating [256]. Clinical experiments have demonstrated that the conjugated linoleic acid found in safflower oil can successfully reduce body fat and weight. Safflower oil has also been discovered to be efficient in reducing fat-induced resistance to insulin, a prevalent issue [257,258].

### 5.8. Sunflower Oil

Sunflower contains manganese, vitamins, phytosterols, dietary fiber, tocopherols, triterpene glycosides, the antioxidant phenolic acids, peptides, flavonoids, carotenoids, caffeine, tannins, chlorogenic acid, saponins, and alkaloids, all of which are beneficial and help in functional growth. The primary healthy components of sunflower seeds and oils—which include high levels of proteins, polyunsaturated and monounsaturated fats, tocopherols, phytosterols as copper, folate, vitamin B, iron, and zinc with anti-diabetic, antihypertensive, calming, anticancer, and antibacterial capabilities—have been linked with the health benefits of these foods [259].

### 5.9. Sour and Sweet Cheery Oil

Sour cherry kernel extract lessens the damage caused by ischemia and regeneration in separated rat hearts. The bioactive chemicals in the kernels of bitter cherry trees may be responsible for this protective effect. These and hydroxycinnamates are present in the kernels [260]. Additionally, according to reports, sour cherry kernels contain 32–36% oil, which is high in α-sitosterol, β-tocopherols, and saturated fatty acids, particularly oleic linoleic acids, among others [261].

### 5.10. Pumpkin Oil

Rich in bioactive substances, pumpkin seeds have been utilized repeatedly for use in meals or medications. The oil from pumpkin seeds has been linked to several health advantages, including avoiding the development of prostate disease, delaying the progression of hypertension, lowering urethral and bladder pressure and enhancing bladder conformity, mitigating hypercholesterolemia and arthritis, decreasing the incidence of breast, colorectal, lung and gastric cancers, having a powerful antioxidant effect, and reducing the risk of diabetes by promoting hypoglycemic activity [262].

### 5.11. Pomegranate Oil

Depending on the cultivar, pomegranate fruits can have between 40–100 g of seeds per kilogram of fruit [263]. The cold-pressed grape seed oil has much better antioxidant qualities than red wine, tea made from green tea, and the artificial antioxidant butylated hydroxy anisole (BHA). The oil from pomegranates contains peculiar trans-18-carbon fatty acids, also known as punicic acids, ranging from 45 to 70% in content [264]. An excellent source of omega-3 fatty acids can also be found in pomegranate seed oil. Fish is one of the most effective sources of n-3 polyunsaturated fatty acids with long chains (LCPUFA), which are proven to be beneficial for human health [265].

### 5.12. Palm Oil

The palm fruit is regarded as an ideal food because it contains a variety of vital nutrients. It also has numerous health advantages because it is a rich source of secondary components, some important vitamins, minerals, and dietary fibers [266]. Cinnamic acids and coumaric acids, including some of their derivatives such as vanillic, sinnapic, gallic, syringic, and caffeic acids, are the primary phenolic compounds found in date fruits. Antioxidants also have a significant impact on the well-being of people, since they lower the chance of serious chronic illnesses, such as various cancers, cardiovascular disease, and neurological disorders [267].

### 5.13. Avocado Oil

Condensed tannins (procyanidins type-A and type-B), flavonoids, and phenolic acids were the most represented groups in the samples, according to other researchers who obtained avocado seed oil to determine its chemical makeup [268]. These findings show that the seeds of avocados are a good source of bioactive compounds with significant antioxidant activity. Moreover, avocado seed oil has more nutrients than other seed oils with higher levels of saturated fats due to its good fatty acid composition and high oleic acid content [269,270].

### 5.14. Chia Oil

Chia is a botanical name for *Salvia hispanica*, an herbaceous plant in the Lamiaceae family. Minerals (4.38%), protein (15–25%), dietary fiber (34–37%), and naturally occurring antioxidants like phytosterols, polyphenols, tocopherols, and carotenoids are all predominantly derived from chia seeds [270]. Because they contain a greater quantity of phenolic components, chia seeds are believed to have exceptional antioxidant effects [271]. The seeds of chia are also recognized as a crucial source of alanine that is usually found in food. Chia seeds are available in a variety of types, such as gel, flour, and raw, incorporated in food that includes dairy and baked commodities [272]. Because of its potent water-absorbing and gel-forming abilities, it is also commonly employed in the pharmaceutical sector as an emulsifier and adjuster [273].

### 5.15. Kangar Oil

The generic name for Kangar is *Gundelia tehranica*, and it is frequently grown in the Middle East. This plant’s different components are widely utilized in both traditional medical use and food [274]. Many studies have shown the medicinal benefits of Kangar seed oil, involving antibacterial capacity, hypoglycemic potential, antioxidant properties, hepatoprotective abilities, and anti-inflammatory potential [275]. In oil derived from several Kangar plant components, fatty acids, including oleic, palmitic, and linoleic acids, have been identified in considerable quantities [276].

### 5.16. Sacha Inchi Oil

The native plant *Plukenetia volubilis*, which is nurtured abundantly in the Peruvian rainforests and is a member of the Euphorbiaceous family, is also referred to as sacha inchi and “Inca peanut” [277]. The main raw material of sacha inchi oil is in its seeds, which comprise a substantial quantity of polyunsaturated fatty acids (85%), having an especially high percentage of linoleic (34%) and linolenic (51%) acids [278,279].

### 5.17. Pistachio Oil

Iran natively cultivates the plant variety *Pistacia khinjuk*, which is a member of the Anarcardiaceae family of plants [280]. This plant’s several components have been demonstrated to exhibit antidiabetic properties, anticancer properties, and advantageous impacts on cholesterol levels and the activity of the liver [277]. In earlier times, it was additionally utilized for medicinal purposes in Iran to alleviate diarrhea, vomiting, and nausea. Many researchers examined the oxidative durability, chemical makeup, and antioxidant action of the kernel and hull of pistachio oil [281].

### 5.18. Cotton Oil

The result of cotton ginning is cottonseed, which contains 16–17% of the oil found in cottonseeds by weight. A small percentage of numerous substances, comprising sterols, phospholipids, tocopherols, resins, carbohydrates, insecticides, gossypol, and additional colors, are found in the unsaponifiable fraction of the oil produced from cottonseed. Shortening and margarine are frequently produced with it as a liquid oil [282]. The oil obtained from cottonseeds is utilized for many different kinds of purposes, like making rubber, processing lubricant sulfonated oil, making soap, processing fabrics, making pharmaceuticals, leather goods, plastic made from synthetic materials, polishing agents, resin, and printing inks in very small quantities [283].

**Table 2:** Major and minor components in oils and their health benefits.

Plant Oil	Fatty Acid Profile	Bioactive Compounds	Vitamin Contents	Health Benefits	References
Chia oil	61% α- Linolenic acid, Oleic acid, palmitic and stearic acids, linoleic acid,	Caffeic acid, chlorogenic acid, querencetin, rosmarinic acid, gallic, cinnamic, myricetin, kaemferol, isoflavones, such as daidzein, glycitein, and genistein,	Vitamin B1 (0.6 mg/100 g), vitamin B2 (0.2 mg/100 g), and niacin (8.8 mg/100 g)	Anti-inflammatory effect, improves cardiovascular disease, decreases cancer risk	[284]
Palm oil	Oleic acid, linoleic acid, and Palmitic acid in a ratio of 43%, 11%, and 40% also lauric 22%, myristic 10%, and stearic acid 3%	The highest amount of catechin, vanillic acid, luteolin, tyrosol, anthocyanins, and carotenoids	Vitamin E in the form of tocotrienols and tocopherols	Cardiovascular and inflammatory diseases, obesity, and some cancers	[285]
Sesame oil	82% unsaturated fatty acids along with a balanced amount of linoleic and oleic acid in sesame seed oil	Sesame seeds contain the lignans sesamolin, sesamin, pinoresinol, and lariciresinol	Sesame oil is full of antioxidants. Along with vitamin E and phytosterols	Lower blood cholesterol and lipid levels, provide anti-inflammatory properties, increase hepatic mitochondrial and neuroprotective effects on brain damage or hypoxia	[286]
Olive oil	7.5 to 20.0% palmitic acid, 0.5 to 5.0% stearic acid, 55.0 to 83.0% oleic acid, 3.5 to 21.0% linoleic acid	Contains small quantities of free fatty acids (FFA), glycerol, phosphatides, pigments, flavor compounds, sterols, and microscopic bits of olive	Contains a modest amount of vitamins E and K	Anticancer, anti-angiogenic, and anti-inflammatory properties	[287]
Eucalyptus oil	6% α-phellandrene, 12% aromadendrene, 6% α-pinene, 5% globulol, ledene, P-cymen, and β-pinene	Eucalyptol, citronellal, citronellol, citronellyl acetate, p-cymene, eucamalol, limonene, linalool, α-pinene, γ-terpinene	Significant amounts of vitamin B1 with appreciable amounts of vitamins B2, B9, and C	Antibacterial, antiseptic, antioxidant, anti-inflammatory, and anticancer activities	[22,229,231]
Sunflower oil	Polyunsaturated fatty acids, particularly linoleic acid, monounsaturated fatty acids, especially oleic acid, 15% saturated fatty acids, particularly stearic acid and palmitic acid	Sterol is found in a concentration of 0.24 to 0.26%	Excellent source of vitamin E, providing about 7.4 mg or just under 50% of the daily value set by the FDA	Control cholesterol level, anticancer effect, antifungal action, antidiabetic properties	[69]
Safflower oil	6–8% palmitic, 2–3% stearic, 16–20% oleic, and 71–75% linoleic acids	Pinoresinol, vanillin, rutin, trans-chalcone, naringin, and tyrosol	Safflower seed oil had α-tocopherol at 376.6 mg/kg, as the main component also contains fat-soluble vitamins (A, D, E, and K)	Controlling blood parameters and cholesterol levels, preventing bone loss in osteoporosis, safflower oil handled skin issues and acne vulgaris	[82]
Linseed oils	57 to 76% of polyunsaturated fatty acids, especially 52% to 60% α-linolenic acid (ALA), 5–6% palmitic acid, 4–5% stearic acid, and 15–20% oleic acid	Manganese, magnesium, phosphorus, and copper	Vitamin B1 (thiamine)	Prevent cardiovascular diseases, antidiabetic, treatment of immune disorders	[82]
Grape oil	85–90% polyunsaturated fatty acids, 60 to 70% of linoleic acid, oleic acid also present in lesser amount	Catechins, epicatechins, trans-resveratrol, and procyanidin B1	Tocopherols and antioxidants	Anticancer activity, antioxidant activity, the role of grape seed oil in cell cycle control, antimicrobial and anti-inflammatory activity	[288,289]
Camelina oil	60% of polyunsaturated fatty acids along with 35 to 40% α-linolenic acid, 16.1% of oleic acid, 1.4% saturated fatty acids	Protocatechuic acid, catechin, sinapine, ellagic acid, sinapic acid, rutin, quercetin-3-O-glucoside, glucosinolates, phytic acid, and condensed tannins	Good source of vitamin E and a-tocopherol	Anticancer activity, prevention from cardiovascular diseases, hypertension, and diabetes	[290,291]
Flax oil	9–10% of saturated fatty acids (palmitic and stearic), about 20% monounsaturated fatty acids (mainly oleic acid), and more than 70% α-linolenic fatty acids	Ferulic acid, Chlorogenic acid, Gallic acid, Secoisolariciresinol, Limamarin, Laricinesol, Linustatin, Pinoresinol, Neolinustatin, and flavonoids	Niacin, riboflavin, folate, vitamin B6, B12, and vitamin C	Prevention from chronic, cardiovascular, and obesity disorders, and cancer	[292,293]
Avocado oil	42% oleic acid, 8% plamitoleic acid, 18% linoleic acid, and 34%, palmitic acid	Condensed tannins (procyanidins type-A and procyanidins type-B), phenolic acids, and flavonoids	150.6–265.75 mg ascorbic acid and 1.92 mg antioxidant	Inhibit the synthesis of fatty acids and triglycerides in the liver, also inhibit apolipoprotein B in VLDL, control the cholesterol level (in animals) and prevent coronary heart disease (both in animals and humans)	[159,162]
Papaya oil	Oleic 74.2%, palmitic 14.9%, stearic 5.2%, and linoleic acid 3.5%	Benzyl isothiocyanate (BITC)	Vitamins A, C, and E	Anticarcinogenic activity, decrease cholesterol concentrations, and prevent coronary heart disease	[215]
Pomegranate oil	36.0% Punicic acid, 43.9%, oleic, 6.2% linoleic 5.5%, stearic acids, and palmitic 2.4%	363.6 to 552.7 mg/100 g Sterols and antioxidants	Vitamin E	Anticancer activity (in vitro)	[294,295]
Pumpkin oil	73.7% unsaturated fatty acids and 25.0% saturated fatty acids (Linoleic 47.5%, palmitic 17.6%, oleic 25.5%, stearic 7.6%, and linolenic acid 0.7%)	Phytosterols, mainly 24 S ethyl 5 alphacholesta 7, 22E-dien-3 betaol (α spinasterol)	Vitamins tocopherol, carotenoid, vitamins A, D, and K	Prevention or alleviation of prostatic hypertrophy (in animals); phytosterols can not only reduce the prostate mass but also inhibit the synthesis of proteins in the prostate	[296,297]
Sweet & sour cherry oil	83.81% unsaturated fatty acids such as linoleic acid 41.5%, oleic acid 35.1%, nervonic acid 4.0%, and eico-sapentenoic acid 2.9%, and 12.2% saturated fatty acids such as stearic acid 3.0% and palmitic acid 9.1%	Antioxidants and polyphenol contents 6.28 mg GAE/kg oil	428.62 mg/L tocopherols and β-carotene 8.47 mg/L	Control the cholesterol level and prevent cardiovascular diseases	[298,299]
Kangar oil	Linoleic acid 572.9 ± 4.9 g kg^−1^, oleic acid 248.4 g kg^−1^ palmitic acid 97.2 g kg^−1^, and unsaturated fatty acids	Conjugated dienes, conjugated trienes, carbonyl and anisidine	Tocopherols and antioxidants	Antibacterial, anti-inflammatory, hypolipemic prospective, and hepatoprotective activity	[300,301]
Sacha inchi oil	Polyunsaturated fatty acids (85%) such as 33.5% of linoleic acid and 44% of linolenic acid	Terpenoids, saponins, and phenolic compounds (flavonoids)	Vitamin E, polyphenols, and minerals	Antibacterial, anti-inflammatory, skin tightening, anti-aging effects, and anticancer	[302]
Pistachio oil	Palmitic acid 19.44%, linoleic acid 13.57%, and oleic acid 63.55%	Polyphenols, antioxidants, and flavonoids	Vitamin E (tocopherols) and higher concentrations of natural antioxidants such as tocotrienols	Anticancer, antidiabetic, lowers cholesterol level, and improvement in liver functions	[303,304]
Cotton oil	Linoleic acid 54.4%, oleic acid 18.6%, and palmitic acid 21.6%	Sterols, resins, phospholipids, pesticides, carbohydrates, and gossypol	Flavonoids, 70% tocopherols	Anti-inflammatory and cardio-protective properties	[283]

## 6. Therapeutic Properties and Functional Applications of Seed Oils

**Figure 1:**
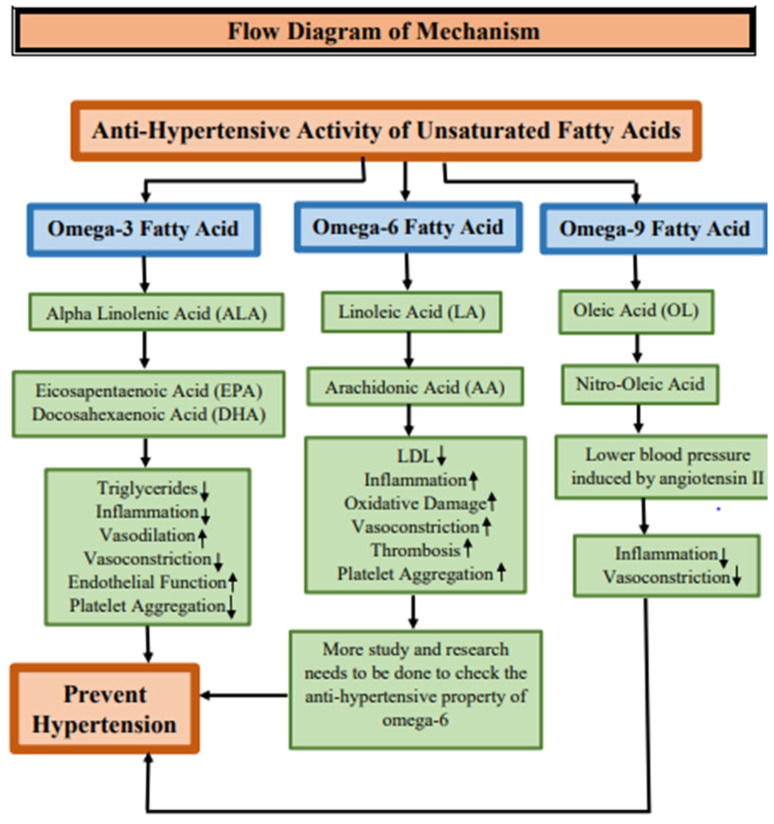
Flow diagram mechanism of antihypertensive activity of unsaturated fatty acids (Downward arrows indicate decreasing concentration, while upward arrows indicate increasing concentration).

The therapeutic properties of the oils have been briefly described in Table 3. Omega-3 fatty acids are essential fatty acids that provide multiple health benefits. A meta-analysis with 387 participants was conducted to check the effects of omega-3 supplementation that resulted in no significant reduction in triglyceride levels and LDL levels of patients with metabolic syndrome and a significant reduction in serum triglycerides levels, systolic blood pressure, and diastolic blood pressure of patients with metabolic syndrome [47]. Another meta-analysis with 25 participants was conducted to evaluate seafood derived omega-3 PUFA levels and their role in chronic kidney disease (CKD), and concluded that higher seafood derived omega-3 PUFA levels were associated with lower risk of incident CKD, although this association was not found for plant-derived omega-3 PUFA [48]. Another research study with 242 pregnant women was conducted and results suggested that women with higher levels of red blood cells (RBC’s) n-3 PUFA status during early pregnancy may be at lower risk for depression at 12 months postpartum [49] (Figure 1).

Omega-6 fatty acids are essential fatty acids that give numerous health benefits. Research was conducted to check the association between n-6 and cardiovascular disease; and concluded that omega-6 PUFA supplementation did not affect the risk for CVD morbidity and mortality but decreased the levels of serum total cholesterol and lowered the LDL levels from the blood which directly related with myocardial infarction and coronary heart disease, which leads to CVD, but further research is needed to elucidate the effects of omega-6 PUFAs on cardio-metabolic outcomes [50]. Another trial with 62 participants was conducted to identify the significance of n-6 PUFA between air pollutants and lung health. The authors concluded that people taking n-6 PUFA supplementation have a low incidence of lung infection as compared to other groups with no n-6 supplementation, and it also improves overall lung health by reducing oxidative stress in the capillary exchange system [52].

These days people are taking more interest in medicinal plants than pharmaceuticals to prevent or cure various discomforts. Medicinal plants are well known for their medicinal properties because of the presence of nutraceuticals [305]. Nutraceuticals are compounds that are not nutrients but act as immune nutrients and protect the human body from pathogen attacks by acting as antioxidants, anti-inflammatory, anticancer, analgesics, agonists, antimicrobial, and provide multiple other benefits [306]. The WHO (World Health Organization) estimated that 80% of the population worldwide is benefited more from nutraceuticals than pharmaceutics [307,308].

Lemongrass oil, citronella oil, and palmarosa oil have medicinal active compounds that demonstrated anticancer and antimicrobial properties. Flaxseed oil acts as a functional food due to its high value of omega-3 acid, lignin (fiber), and lignans (phytoestrogens), which act strongly in regard to cardiovascular health and lower serum cholesterol levels. Evening primrose oil is rich in gamma-linolenic acid (omega-6) and provides 100% of the recommended dietary allowance (RDA) of vitamin E; it benefits polycystic ovarian syndrome (PCOS), lowers blood cholesterol, reduces inflammation in arthritis, and promotes heart, hair, and skin health [45].

**Figure 2:**
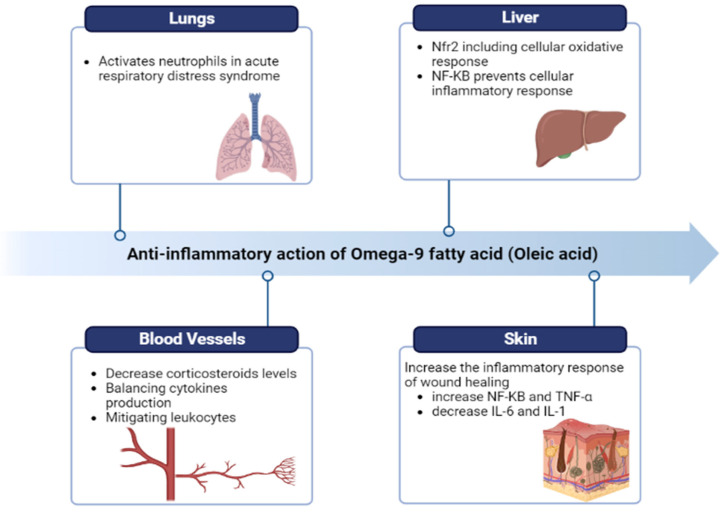
The anti-inflammatory action of omega-9 fatty acids (ω-9 fatty acids) in lungs, liver, blood vessels, and skin.

Industrial oils are highly oxidative due to extreme processing, which can be adverse for human health. High inflammation-rated industrial oils include sunflower, safflower, grapeseed, soybean, corn, canola, and rice bran oils. During the extraction process, seeds are heated at high temperatures that oxidize their fatty acids producing free radicals. These oils when combined and termed as vegetable oil with the fortification of omega-6 causes toxicity of omega-6 in oil; when humans consume these oils rich in omega-6, it triggers the body to produce pro-inflammatory chemicals and also disturbs the omega-3:6 balance in the body. Industrial oils are not rich in nutrients like vitamins E, D, A, and K, while they are rich in polyunsaturated fat that affects the heart and liver by causing atherosclerosis, arteriosclerosis, and fatty liver, and these conditions can lead to heart failure/heart attack, obesity, and liver cancer [46,309] (Figure 2).

**Table 3:** Fatty acids and their therapeutic properties.

Fatty Acids and Their Types	Seed Sources	Other Sources	Therapeutic Uses	Recommended Dietary Allowance (RDA)/Day	References
Omega-3 (ALA-alpha linolenic acid, EPA-eicosapentaenoic acid, and DHA-docosahexaenoic acid) Essential fatty acids	Chia seed oil, linseed oil, walnut oil, borage oil, evening primrose oil, olive oil, flaxseed oil, navy bean oil, and pecan oil	Cold water salmon, leafy green vegetables, winter squash, kidney beans, tofu, oysters, seafood, brussels sprouts, muskmelons, berries, and avocados	Improving heart health by managing cholesterol, triglycerides, and blood pressure levels, supporting mental health, builds cellular membranes in brain, prevent depression, help in weight reduction especially waist size, lowering liver fat, supporting infant brain development, preventing blood clotting, and fighting inflammation	Males: 1.6 g Females: 1.1 g	[307,310]
Omega-6 (linoleic acid, GLA-gamma-linolenic acid, AA-arachidonic acid, and CLA-conjugated linoleic acid) Essential fatty acids	Grapeseed oil, corn oil, walnut oil, cottonseed oil, soybean oil, sesame oil, peanut oil, olive seed oil, borage oil, evening primrose oil, almond oil, flaxseed oil, pistachio oil, pecan seed oil, sunflower oil, and pumpkin seed oil	Duck fat, chicken fat, bacon grease, goose fat, eggs, popcorn (air popped), corn, chicken liver, cooked carrots, beef tallow, butter, brown rice, beef liver, and whole wheat flour	Reduces inflammation, reduces excessive fat mass from organs at cellular levels, play key role in immune system by boosting immunity and keeps immune cells safe from foreigners, regulates brain functioning, regulates growth and development, maintains reproductive system, and maintains healthy bones	Males = 17 g Females = 12 g	[311,312]
Omega-7 (Palmitoleic acid, rumenic acid, and vaccinic acid) Non-essential fatty acids	Macadamia nuts (seed oil) and sea buckthorn seed oil	Salmon, anchovies, and avocado	Lipokine activity improves digestion, improves liver health at the cellular level, boosts collagen production, influences healthy fat metabolism, encourages healthy eye lubrication and tear production, and reduces insulin resistance	About 2.5 g	[313,314,315]
Omega-9 (OA-Oleic acid) Non-essential fatty acids	Olive seed oil, cashew nut oil, almond oil, and peanut oil	Avocado, animal liver, and seafood	Improved insulin sensitivity, decreased inflammation, and improves joint health and healing	---	[316,317,318]

## 7. Cosmetics Properties

**Figure 3:**
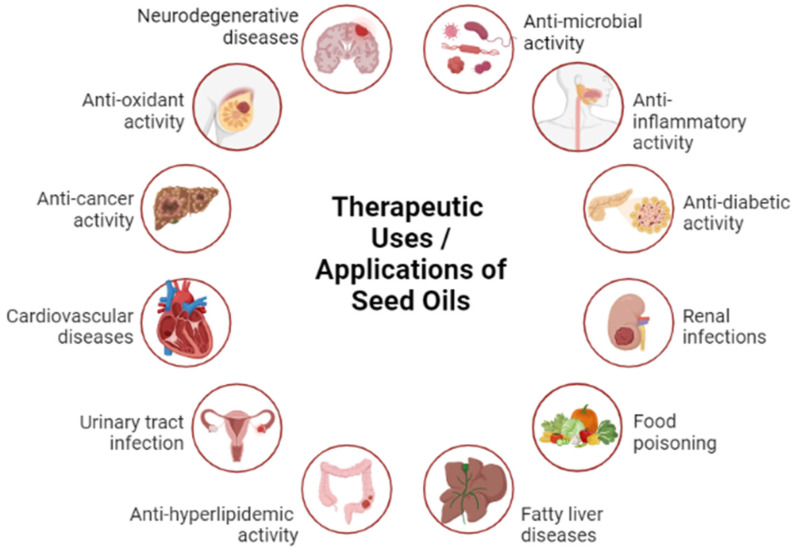
Therapeutic uses and applications of seed oils.

Fats and oils are important components of cosmetic formulations. They provide moisturizing, emollient, grooming, and skin conditioning effects to skin care products. The cosmetic industry is a multibillion-dollar business and has always been growing interest, especially towards natural product-based ingredients instead of synthetic chemicals incorporated in skin care products. Oxidative stress is the major cause of skin-related problems and also contributes to rapid aging processes [315,319]. Seed oils like castor oil are one of the most important and beneficial products in skin and hair health and it is used in pure form as a product. Sunflower seed oil contains linoleic acid and essential fatty acids, which activates the peroxisome proliferator-activated receptor-alpha (PPAR-alpha) that stimulates keratinocyte differentiation and lipid metabolism and improves barrier function in skin cells. These properties make it important as a therapeutic formulation ingredient in anti-aging skin care products [320]. Details on this subject are presented in Table 4 and Figure 3.

## 8. Contraindications of Omega (ω) Fatty Acids

Overconsumption of plant seed oils high in omega-3 and omega-6 fatty acids may have detrimental effects; however, omega-3 fatty acids have fewer negative effects than omega-6 fatty acids. These adverse effects could vary based on the dosage, the duration, and the person’s sensitivities to omega-3 and omega-6 fatty acids. The recommended amount of omega-6 fatty acids per day is 10 g. Additionally, some professionals assert that the ideal ratio of omega-3 to omega-6 fatty acids is roughly 4:1 [330]. These side effects are caused by excessive omega-3 and omega-6 fatty acids, which adversely affect the body’s inflammatory and blood coagulation systems. To regulate blood clotting, the body produces thromboxane and prostaglandin from omega-3 and omega-6 fatty acids. Reduced levels of these molecules may cause problems with bleeding since they make the blood thinner and inhibit the production of clots. However, when these molecules are high, it can increase the chance of heart disease and stroke as a result of thicker, more clotting-prone blood [315,318].

Excessive consumption of omega-3 fatty acids can cause some unwanted effects such as bleeding gums; coughing; coughing up blood; difficulty with breathing; dizziness; fast or irregular heartbeat; headache; hives; itching or skin rash; increased menstrual flow; abnormal vaginal bleeding; nosebleeds; paralysis; prolonged bleeding from cuts; puffiness or swelling of the eyelids or around the eyes, face, lips or tongue; red or black stools; red or dark urine; excessive night sweating; tightness in the chest; unusual tiredness or weakness; acidic or sour stomach; bad, unusual, or unpleasant taste; belching; bloating; indigestion; stomach discomforts or aches; diarrhea; loss of appetite; flatulence; and difficulty in having a bowel movement [317].

Omega-3 and omega-6 fatty acids can also be used to create other metabolites, including resolvins, protectins, and maresins, which have anti-inflammatory and antitumor characteristics. Among other things, these metabolites can influence the behavior of various immune and epithelial cells by reducing the generation of inflammatory cytokines, enhancing the healing process of inflammation, promoting tissue repair, and inhibiting tumor formation. But additional research is needed to determine the particular pathways through which these metabolites influence skin disorders [331,332,333].

## 9. Conclusions

This review has highlighted the significance and popularity of oils and their components both in therapeutic uses, prevention and cure of various chronic diseases, and as an active ingredient in fragrance and cosmetic industries. Oils exhibit strong therapeutic potential, such as high antioxidant properties, high antimicrobial, and antiviral properties over a wide range of spectrums and proves to be helpful in pathological therapies. Oils play an important role as an integral part in the food and cosmetic industries as they contain a unique and wide range of bioactive compounds that may prevents aging and offer protection from the sun and different skin diseases. These oils and their compounds may be used in the future with more favorable effects in the medical or pharmaceutical industries.

## Figures and Tables

**Table 4:** Seed Oils and their applications in the cosmetic industry.

Seed Oil	Active Constituents	Cosmetic Applications	References
Argon oil	Phenolics, vitamin E, fatty acids (linolenic acid, palmitic acid, linoleic acid, oleic acid, and stearic acid)	Wound healer, emollient, anti-aging, and hair oils (hair nourishing)	[321]
Baobab oil	Rich in vitamins D, E, and A, and a natural source of D3	Bath oil preparations, moisturizer, massage oil, hot oil soaks for hair, and nail conditioning	[322]
Tea seed oil	Richest oil in polyphenols	Hair products (related to some specific hair problems like dandruff, coiled hairs, and split ends) and strong anti-aging ingredient	[323]
Hemp oil	The highest content of omega-3 and -6 fatty acids	Skin regenerative, in ointments/creams related to eczema and acne, and anti-aging	[324]
Kalahari melon oil	Rich in E vitamin	Light skin moisturizer, skin regeneration, and foaming agent to treat skin discoloration/tanning and acne/vulgaris	[325]
Black cumin seed oil	Linoleic acid, phytosterols, and thymoquinone	Beneficial for acne, hair loss, and toothache	[165]
Date seed oil	Phenolic, PUFA, and carotenoids	In shaving soaps, body creams, shampoos, and sunscreen (protective mechanism against UV-B and UV-A lights)	[326]
Cranberry seed oil	Omega-3, -6, and -9 fatty acids, PUFA	The highly moisturizing effect, hand and body creams, and hair shampoo	[327]
Jojoba oil	Wax esters of fatty acids	Hair moisturizer	[328]
Grape seed oil	PUFAs, linoleic acid, vitamin E, proanthocyanidins, and polyphenols	Light oil, skin moisturizers, acne products, skin lightening agent, promotes hair growth, and anti-aging	[329]

## Data Availability

Data are available from the corresponding author upon request.

## Abbreviations

Accelerated solvent extraction (ASE), Alfa (α), Alpha-linolenic acid (ALA), Angiotensin-converting enzyme (ACE), Arachidonic Acid (AA), Arachidonic acid (AA), Atomic fluorescence spectroscopy (AFS), Benzyl isothiocyanate (BITC), Butylated hydroxy anisole (BHA), Carbon dioxide (CO_2_), Cardiovascular disease (CVD), Chronic kidney disease (CKD), Cold-pressed extraction (CPE), Conjugated linoleic acid (CLA), Coronary heart disease (CHD), 2,2-Diphenyl-1-picrylhydrazyl (DPPH), Docosahexaenoic Acid (DHA), Eicosapentaenoic Acid (EPA), Electron microscope (EM), Enzyme-assisted extraction (EAE), Flame ionization detector (FID), Food and Drug Administration (FDA), Fourier transform infrared spectroscopy (FTIR), Free fatty acids (FFA), Gallic acid equivalent (GAE), Gamma-linolenic acid (GLA), Gas chromatography (GC), Gas chromatography–infrared spectroscopy (GC-IR), Gas chromatography–mass spectrometry (GC-MS), Gas chromatography (GC), Gel permeation chromatography (GPC), Gel permeation chromatography infrared spectroscopy (GPC-IR), High-density lipoprotein (HDL), High-performance liquid chromatography (HPLC), High-resolution gas chromatography (HRGC), High-resolution mass spectrometry (HRMS), Laser diffraction (LD), Limit of detection (LOD), Limit of quantification (LOQ), Linoleic Acid (LA), Liquid chromatography (LC), Liquid chromatography–mass spectrometry (LC-MS), Long chain n-3 polyunsaturated fatty acids (LCPUFA), Low-density lipoprotein (LDL), Low-molecular weight (LMW), Mass-to-charge ratio (*m*/*z*), Medium-chain fatty acids (MCFA), Microwave-assisted extraction (MAE), Million tons (Mt), Molecular weights (MW), Monounsaturated fatty acids (MUFA), Myocardial infarction (MI), Oleic Acid (OA), Oleoyl dilinolein (OLL), Omega (ω), Peroxisome proliferator-activated receptor-alpha (PPAR-alpha), Polycystic ovarian syndrome (PCOS), Polyunsaturated fatty acids (PUFA), Pressurized liquid extraction (PLE), Pulsed electric fields-assisted extraction (PEF), Recommended dietary allowance (RDA), Red blood cell (RBC), Saturated fatty acids (SFA), Scanning electron microscopy (SEM), Signal to noise (s/n) ratio, Solid-phase micro extraction (SPME), Solvent-free microwave extraction (SFME), Supercritical-fluid extraction (SFE), Triacylglycerols (TAG), Trilinolein (LLL), Ultrasound-assisted extraction (UAE), Ultraviolet (UV), Unsaturated fatty acids (UFA), Weight (wt.), World Health Organization (WHO).
